# Prediction of hydrophilic and hydrophobic hydration structure of protein by neural network optimized using experimental data

**DOI:** 10.1038/s41598-023-29442-x

**Published:** 2023-02-07

**Authors:** Kochi Sato, Mao Oide, Masayoshi Nakasako

**Affiliations:** 1 Department of Physics, Faculty of Science and Technology, Keio University, 3-14-1 Hiyoshi, Kohoku-ku, Yokohama, Kanagawa 223-8522 Japan; 2 RIKEN SPring-8 Center, 1-1-1 Kouto, Sayo-cho, Sayo-gun, Hyogo 679-5148 Japan; 3 PRESTO, Japan Science and Technology Agency, Chiyoda-ku, Tokyo, 102-0076 Japan

**Keywords:** Computational biology and bioinformatics, Structural biology

## Abstract

The hydration structures of proteins, which are necessary for their folding, stability, and functions, were visualized using X-ray and neutron crystallography and transmission electron microscopy. However, complete visualization of hydration structures over the entire protein surface remains difficult. To compensate for this incompleteness, we developed a three-dimensional convolutional neural network to predict the probability distribution of hydration water molecules on the hydrophilic and hydrophobic surfaces, and in the cavities of proteins. The neural network was optimized using the distribution patterns of protein atoms around the hydration water molecules identified in the high-resolution X-ray crystal structures. We examined the feasibility of the neural network using water sites in the protein crystal structures that were not included in the datasets. The predicted distribution covered most of the experimentally identified hydration sites, with local maxima appearing in their vicinity. This computational approach will help to highlight the relevance of hydration structures to the biological functions and dynamics of proteins.

## Introduction

Proteins fold into unique structures in water and/or lipid bilayers and conduct biochemical and biophysical processes in the aqueous environment of living cells^1^. Water molecules act as important building blocks for protein structures and molecular interactions in protein–protein complexes^2,3^, as stabilizers for optimizing chemical reactions in enzymes^4^, and as regulators of internal motions for performing biological functions^5–7^. Therefore, structures and interactions at the protein-water interface, the hydration structures of proteins, are subject to discussion for understanding the roles and influences of hydration water molecules on folding, stability, and functions of proteins at the atomic level^8^.

The hydration structures of proteins have been investigated using various biophysical techniques^8–15^. In particular, atomic details of protein hydration, such as the locations and interactions of hydration water molecules, are visualized by high-resolution crystal structure analyses at cryogenic temperatures^6,16^. Cryogenic transmission electron microscopy (cryoTEM)^17^ can be used to study the hydration structures of proteins. In contrast to X-ray crystallography, since protein molecules are flash-cooled for cryoTEM analysis, conformational substates inherently appearing in solution can be observed^18,19^. Therefore, cryoTEM observations may help to elucidate the hydration structure changes among the conformational substates. Unfortunately, even at a resolution where many hydration water molecules are detectable in electron density maps from X-ray crystallography data, fewer molecules are visible in potential maps in cryoTEM analyses^20^, and this may result due to the low scattering cross-section of oxygen atoms for electrons.

In structural analyses, hydration structures of whole protein surfaces are incompletely characterized because of factors such as the positional disorder of hydration water molecules, resolution in structure analyses, and/or molecular contacts in crystals. Therefore, computational approaches are necessary to completely illustrate the hydration structures of the entire protein surface. In our previous study, a set of empirical distributions of hydration water molecules surrounding polar protein atoms were obtained through the database analysis for crystal structures of proteins^21^ and were used to predict the distribution of hydration water molecules in interior cavities and on hydrophilic surfaces of proteins^22,23^.

In addition to the knowledge-based approach, molecular dynamics (MD) simulations have shown the potential to provide structural information on protein hydration at a high spatiotemporal resolution^24–26^. The force field parameters tuned by referring to the empirical distributions reproduced the hydrogen-bond patterns between hydration water molecules and polar protein atoms in MD simulations^27^. In the last two decades, protein hydration has been a subject of statistical mechanical theory for liquids, for instance, the three-dimensional reference interaction site model (3D-RISM)^28^. However, both approaches require large computational times and costs, and the development of the 3D-RISM to reproduce hydration structures over protein surfaces is still in progress^8,29^.

In this study, as an alternative computational approach, we constructed a neural network (NN) for predicting the hydration probability distribution over the surfaces and the interior cavities of proteins. The constructed NN was optimized for experimentally identified hydration structures from protein crystallography as recently reported NN-based hydration prediction methods^30,31^, rather than the method trained by the hydration structures predicted by MD and theoretical calculations^32^. Here, we describe the NN architecture details and demonstrate the performance of predicting hydration structures in the interior, hydrophilic, and hydrophobic surfaces of proteins. In addition, we discuss the characteristics of our method through comparison with the other NN-based methods.

## Results

### Construction of NN

**Figure 1:**
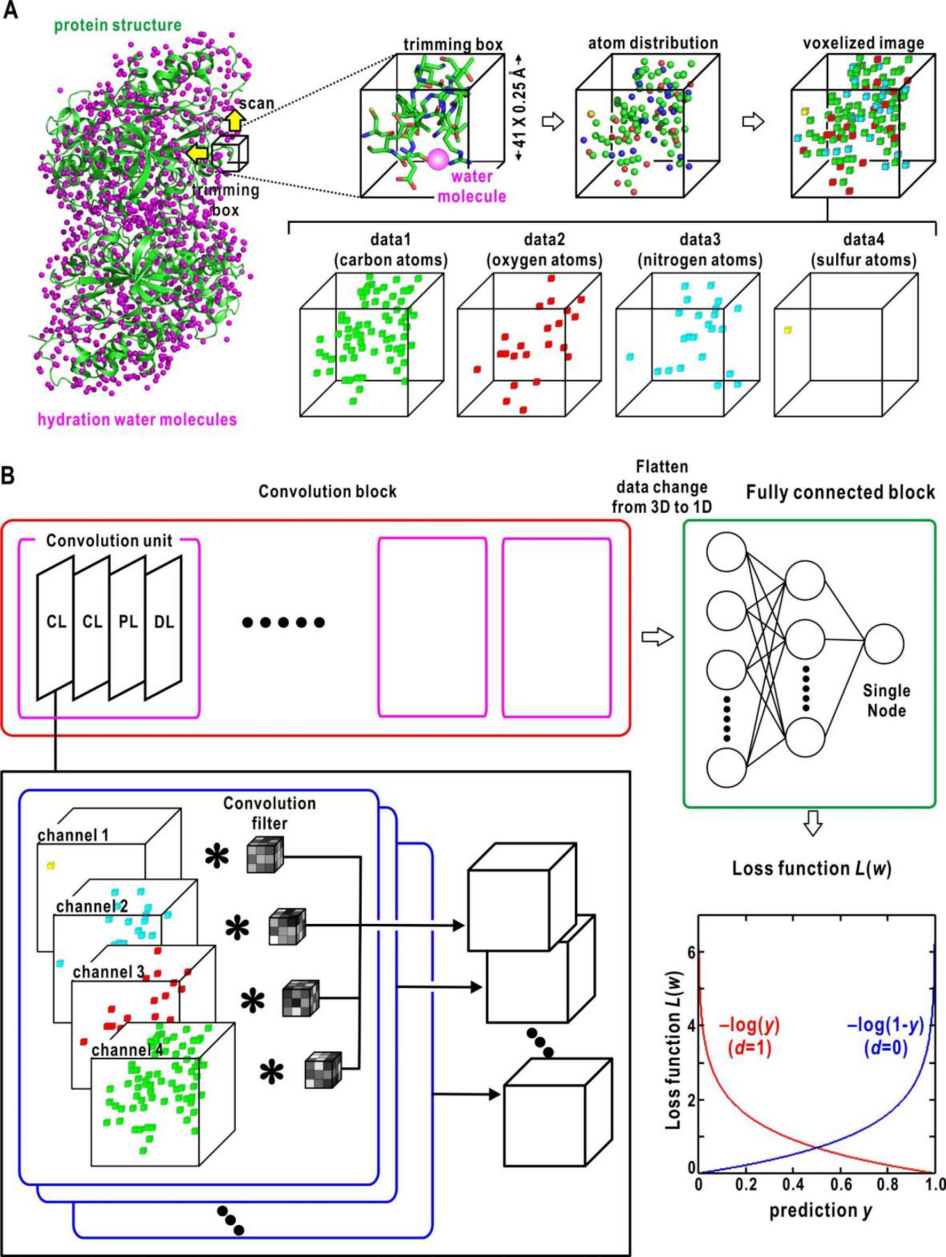
Construction of the NN to predict hydration probability around protein molecules. (**A**) Schematic of the preparation procedure for the dataset from crystal structures of proteins accompanying hydration water molecules. (**B**) Schematic of the NN architecture. The loss function is illustrated in the lower right corner.

Datasets for training the NNs were prepared from 2145 crystal structure models of proteins including 2,655,363 hydration water molecules (Methods section, SI Appendix, S1, S2 and Fig. S1). The dataset provided a set of 5,310,726 voxelized three-dimensional (3D) images of 10.25 × 10.25 × 10.25 Å^3^ regarding the spatial distributions of carbon, nitrogen, oxygen, and sulfur atoms for both 2,655,363 crystal-water present and 2,655,363 crystal-water absent sites (Fig. 1A). Due to the large number of images for the training data, the dataset was used without data augmentation by rotation operation for the images.

Based on the three-dimensional convolutional neural network (3D-CNN), which was composed of a convolution block (CB) and a fully connected block (FCB) (Fig. 1B), six NNs with different CB and FCB architectures were constructed and optimized using the training dataset (the Methods section and SI Appendix, S3). In the previous database analysis^8,21^, we found that the arrangements of protein atoms engaged in protein hydration are predominantly induced to satisfy the tetrahedral hydrogen-bond geometry of water molecules rather than the amino acid sequences of proteins. Therefore, the constructed NNs were trained using the 70% images randomly selected from the datasets, and validated using the 30% images. We selected the most efficient NN to reproduce the hydration sites by inspecting the validation metrics against the validation and test datasets (SI Appendix, S3 and Table S1) and the frequency distribution of hydration probability for the test data (SI Appendix, Fig. S2). For the test data, we used a crystal structure of glutamate dehydrogenase (GDH), which was refined at a resolution of 1.8 Å and included more than 1200 hydration water molecules (SI Appendix, Table S2). The sequence identity of GDH against the 2145 proteins was smaller than 34%. In the selected NN, the CB was composed of two convolution units with 3 × 3 × 3 filter and 32 channels, and the FCB had one layer with 32 nodes (SI Appendix, Model 6 in Table S2).

The NN was further assessed with respect to the reproducibility of hydration structures for the 300 crystal structures of proteins, which were not included in the in the training and validation datasets (Table 1 and SI Appendix, S4). The dataset included 174,796 hydration water molecules, yielding 174,796 crystal-water present and 174,796 crystal-water absent images. The average accuracy and loss scores were 0.873 and 0.298, respectively, and comparable with those for the validation dataset (SI Appendix, Table S1). In addition, the measured precision, recall, and F-score values (Table 1) indicated that the selected NN overpredicted neither the presence nor absence of crystal-water molecules.

**Table 1:** Validation and performance of the selected NN.

	Precision	Recall	F-score	
Validation of the selected NN using a set of 300 crystal structures				
True	0.9010	0.8368	0.8677	
False	0.8476	0.9081	0.8768	
MAD (Å)/RMSD (Å)	0.72/0.98			

GDH/NHase	Number of predicted sites			
	4671/1456			
	First-layer class	Inside class		
Prediction results for GDH and NHase				
Number of crystal-water sites	1425/785	195/170		
Ratio of crystal-water sites with probability greater than 80% (%)	81/72	93/89		
MAD (Å)	0.60/0.68	0.52/0.53		
RMSD (Å)	0.82/0.93	0.73/0.73		

GDH/NHase	Accutar^30^		GalaxyWater-CNN_42^31^	
Number of predicted sites	2751/1327		–*/1027	
	First-layer class	Inside class	First-layer class	Inside class
Prediction results for NHase by the two NN-based prediction methods				
MAD (Å)	1.13/0.72	0.39/0.33	–*/0.70	–*/0.31
RMSD (Å)	1.13/1.10	0.65/0.51	–*/1.10	–*/0.43

*Because of the limitation on the number of residues in the calculation, the hydration prediction for GDH was not executed.

### Predicted hydration probability

**Figure 2:**
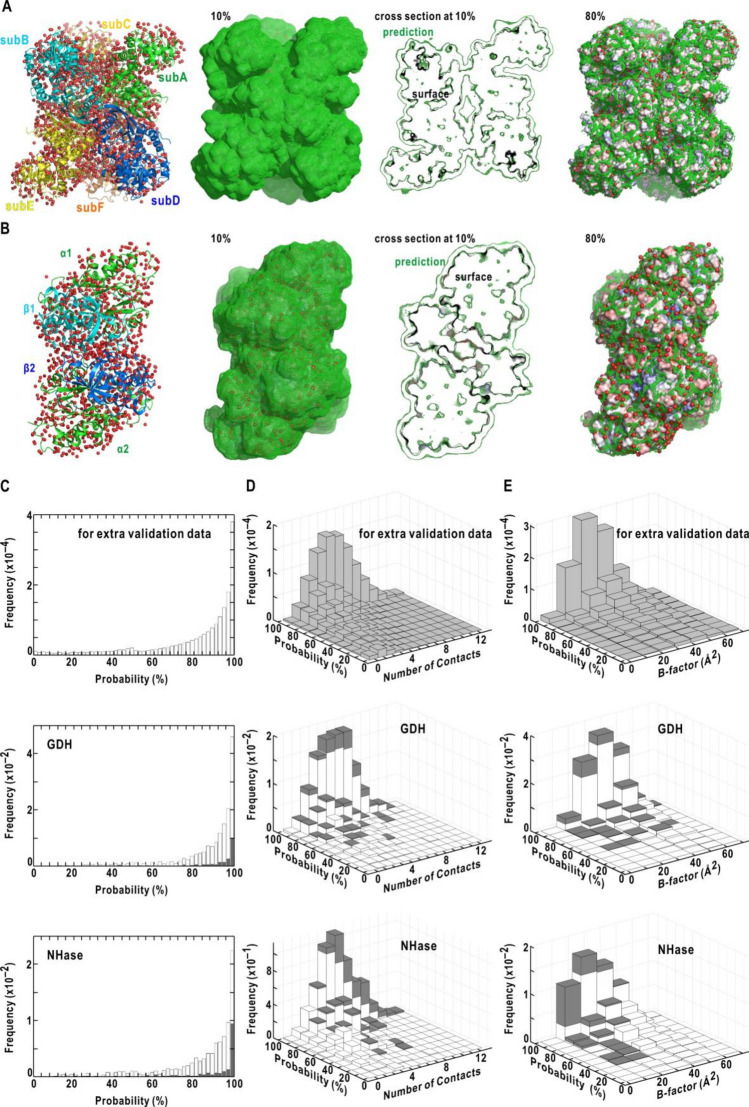
Characteristics of the predicted hydration probability distributions on protein surfaces. (**A**) The predicted hydration distribution for the crystal structures of GDH composed of six subunits (the accession code of PDB: 1euz). (**B**) The predicted hydration distribution for NHase composed of two α-subunits and two β-subunits (the accession code of PDB: 2ahj). The crystal structures of two protein molecules are depicted as ribbon models in the left panels (SI Appendix, Table S2). The probability distributions contoured at 10%, and 80% levels are displayed on the surface-rendered models of the crystal structures. The middle panel is the cross-sectional view of the 10% probability at the plane of the molecular center. Red spheres of 2 Å diameter indicate the locations of crystal-water sites. (**C**) The frequency distributions of the predicted probabilities at crystal-water sites. (**D**) The correlation between the probability and the number of interactions. (**E**) The correlation between the probability and the thermal factors of water molecules. In panels (**C**)**–**(**E**), plot shown are for the 300 protein structures for validation (upper panel), GDH (middle) and NHase (lower). For GDH and NHase, the hydration sites in the first-layer and inside classes are shown using white and gray bars, respectively, and the frequencies of the two classes were stacked in panels (**D**) and (**E**). All molecular images were drawn using PyMOL^60^.

As representative results of the NN-prediction, Figs. 2A and 2B depict the predicted hydration probability distributions for GDH^5,22^ and hetero-tetrameric nitrile hydratase (NHase)^2,33^ (SI Appendix, Table S2), respectively. The predicted hydration probability distribution, contoured at the 10% level, covered the crystal-water sites in the first layer, and the shape of the distribution on the protein surfaces almost reproduced the solvent-accessible surfaces calculated using a sphere with a diameter of 3 Å. At the 80% contour level, which approximately corresponded to the one standard deviation level from the 100% probability in the frequency distribution of the hydration probability in Fig. 2C, the probability distributions were localized around the crystal-water sites. For surfaces suitable for hydration but lacking hydration water molecules in the crystal structures, the probability distributions indicated potential hydration patterns.

For quantitative evaluation, we first inspected the frequency distributions of the predicted hydration probabilities at the crystal-water sites (Fig. 2C and Table 1). For 67% of crystal-water sites in the 300 crystal structures for the validation, the NN yielded the hydration probability greater than 80%. For GDH and NHase, the predicted probability distributions were separately assessed for the crystal-water sites in the first-layer class exposed to bulk solvent and the inside class occupying the cavities and interfaces (Table 1). The hydration structures of both classes are necessary to study the dynamics, stability and intermolecular interactions of the proteins^2–8,22^. The predicted probability was greater than 80% for approximately 90% of the crystal-water sites in the inside class, and the probability for 70–80% of the crystal-water sites in the first-layer class was greater than 80%. The frequency distributions for the inside class were narrower than those for the first layer class, probably because the local maxima of the predicted probability were frequently closer to the crystal-water sites in the inside class than those of the first-layer class as measured by *MAD* and *RMSD* (Table 1).

The predicted probability tended to be higher for hydration sites with a greater number of interactions with protein atoms and adjoining hydration water molecules (Fig. 2D). This tendency was clear in the inside class, where greater number of interactions are expected than the first-layer class. In addition, at the crystal-water sites occupied by unambiguously identified hydration water molecules with the B-factors smaller than 30 Å^2^, the predicted hydration probability tended to be greater than 80% (Fig. 2E). Therefore, based on the frequency distributions shown in Fig. 2C–E, the predicted hydration probability of 80% can be used as a rough threshold to assess the NN-predicted probability distribution.

The quantitative evaluation described above implied that the NN was feasible to predict the hydration structures for the first layer and inside classes. Although the NN was optimized without information on both the interaction energies and positional fluctuations of hydration water molecules, the NN probably learned the tendency underlying the dataset that more interactions with protein atoms ensure a more stable residence of hydration water molecules in hydration sites.

In the following sections, we assessed the performance of the NN by comparing the predicted hydration probability distributions with the experimentally observed hydration patterns of the inside class in cavities and interfaces and the first-layer class on hydrophilic and hydrophobic surfaces.

### Prediction for hydrophilic cavity

Hydrophilic cavities organized in protein interiors and at the interfaces of protein complexes are isolated from the bulk solvent and are filled, in most cases, by hydration water molecules of the inside class^8,34^. The hydration water molecules act as stabilizers for the tertiary and quaternary structures of proteins and display thermal factors comparable to those of the protein atoms in contacts^8^. As protein structural models solved at a low resolution frequently lack hydration water molecules in the inside class, hydration structures in cavities and interfaces must be generated to investigate the structural stability and avoid the artificial collapse of empty cavities during MD simulations under constant pressure.

**Figure 3:**
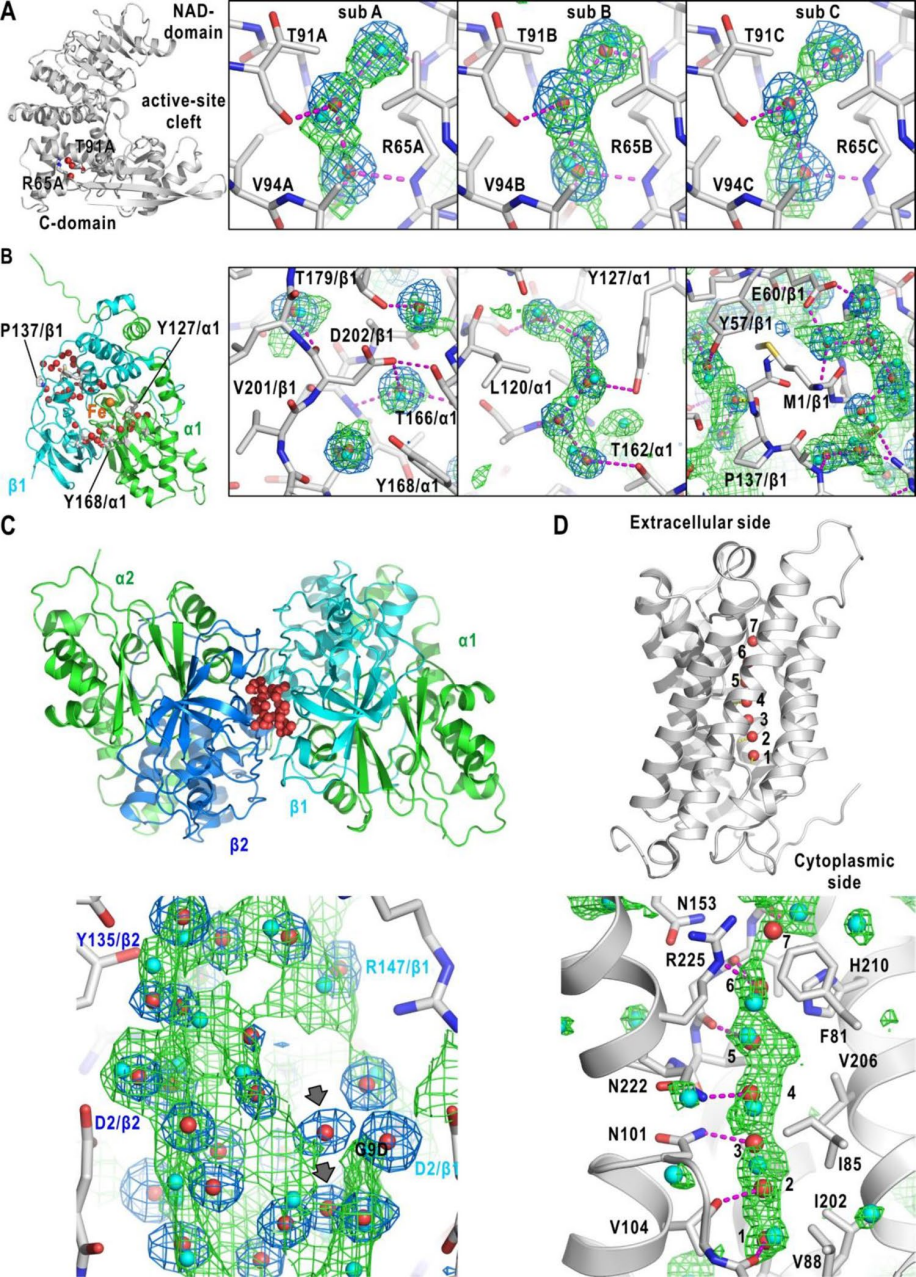
Prediction for cavities, interface, and channel. (**A**) Prediction for the three hydration sites in small cavities in subunits (**A**), (**B**), and (**C**) of GDH. The location of the cavity in subunit (**A**) is illustrated in the left panel. The green mesh is the probability distribution contoured at 80% probability, and the local peak positions are indicated by cyan spheres. The blue nets are the omit-difference Fourier electron density maps of crystal-water molecules (red spheres) at the highest resolution (see Table SI2) contoured at three standard deviation levels from the average. Amino acid residues forming the cavities (or channels) are shown using stick models, and the potential hydrogen bonds are indicated by magenta dashed lines. Illustrations in panels (**B**)–(**D**) are drawn similarly. (**B**) Predicted hydration distributions for three cavities in NHase. The locations of the cavities are shown in the left panel. (**C**) Predicted hydration probability distribution for water molecules confined in the interface between the two β-subunits of NHase (upper panel). The gray arrows indicate the hydration water molecules without direct contact with protein atoms. (**D**) Predicted hydration probability distribution in the solvent channel with seven hydration water molecules (1–7) of a subunit of tetrameric AQP (the accession code of PDB: 1z98, upper panel). Two of the channel-forming α-helices are illustrated as ribbon models.

The NN-predicted hydration probability distributions for protein cavities and interfaces were assessed by inspecting the coverage of hydration sites and the positional differences between hydration sites and local maxima in the distribution. Figure 3A shows the NN-predicted hydration distribution for a small cavity in each subunit of GDH. The cavity is organized by the Arg65, Thr91, and Val94 side chains, and is occupied by three hydration water molecules. The NN yielded similar hydration probability distributions for cavities among the six subunits, and local maxima in the distributions were located in the 0.5–0.7 Å range from the nearest hydration site.

Figure 3B shows the NN prediction for the three cavities in an αβ-heterodimer of NHase. Each of four small cavities surrounded by Thr166/α1, Tyr168/α1, and Asp202/β1 was occupied by a single water molecule. The predicted probability distribution showed four separate peaks that almost overlapped with the four hydration sites. The positional differences between local maxima from the sites were in the 0.3–0.9 Å range. In a cavity formed by Leu120/α1, Tyr127/α1, and Thr162/α1, the predicted distribution covered five hydration water molecules in a zigzag arrangement, and the local maxima were located within 0.4 Å from the sites. The two cavities with bent-tubular shapes surrounded Met1/β. Eight water molecules occupied each cavity. The predicted probability distribution reproduced the distribution of the hydration sites, with the local maxima located in the 0.2–1.1 Å distance range from the nearest sites.

At the interface of two αβ-heterodimers of NHase (Fig. 3C), a cavity of approximately 20 × 12 × 10 Å^3^, a much larger volume than the cavities described above, was filled with 37 hydration water molecules to assist the association of the β-subunits^2^. The NN-predicted probability distribution covered most of the water molecules engaged in direct interactions with protein atoms, and the maxima in the probability distribution were located in the 0.3–1.7 Å range from the nearest hydration sites.

Columnar arrangements of hydration water molecules are found in the interiors of various proteins^22^. A representative example is the tandemly arranged water molecules in the water channel of aquaporin (AQP)^35,36^ (Fig. 3D). The NN-predicted probability distribution covered most of the water molecules in the extracellular vestibule, central region, and cytoplasmic vestibule of the channel. The probability maxima near hydration sites 1, 4, 5, and 6 were located within 0.7 Å from the identified hydration water molecules.

From the prediction results for the cavities and the interface, we hypothesized that the NN can learn the distribution patterns of atoms suitable for hydration in cavities and may be useful for predicting hydration structures in hydrophilic cavities in the interiors of both soluble and membrane proteins.

### Prediction for hydrophilic surface

Hydration water molecules in the first-layer class, each covering an average accessible solvent area (ASA) of approximately 20 Å^2^, are indispensable for the solvation of proteins^8^. Here, we describe how the NN-predicted probability distributions are consistent with the experimental hydration patterns on the complicated surfaces of proteins in the stationary state and those undergoing conformational changes.

**Figure 4:**
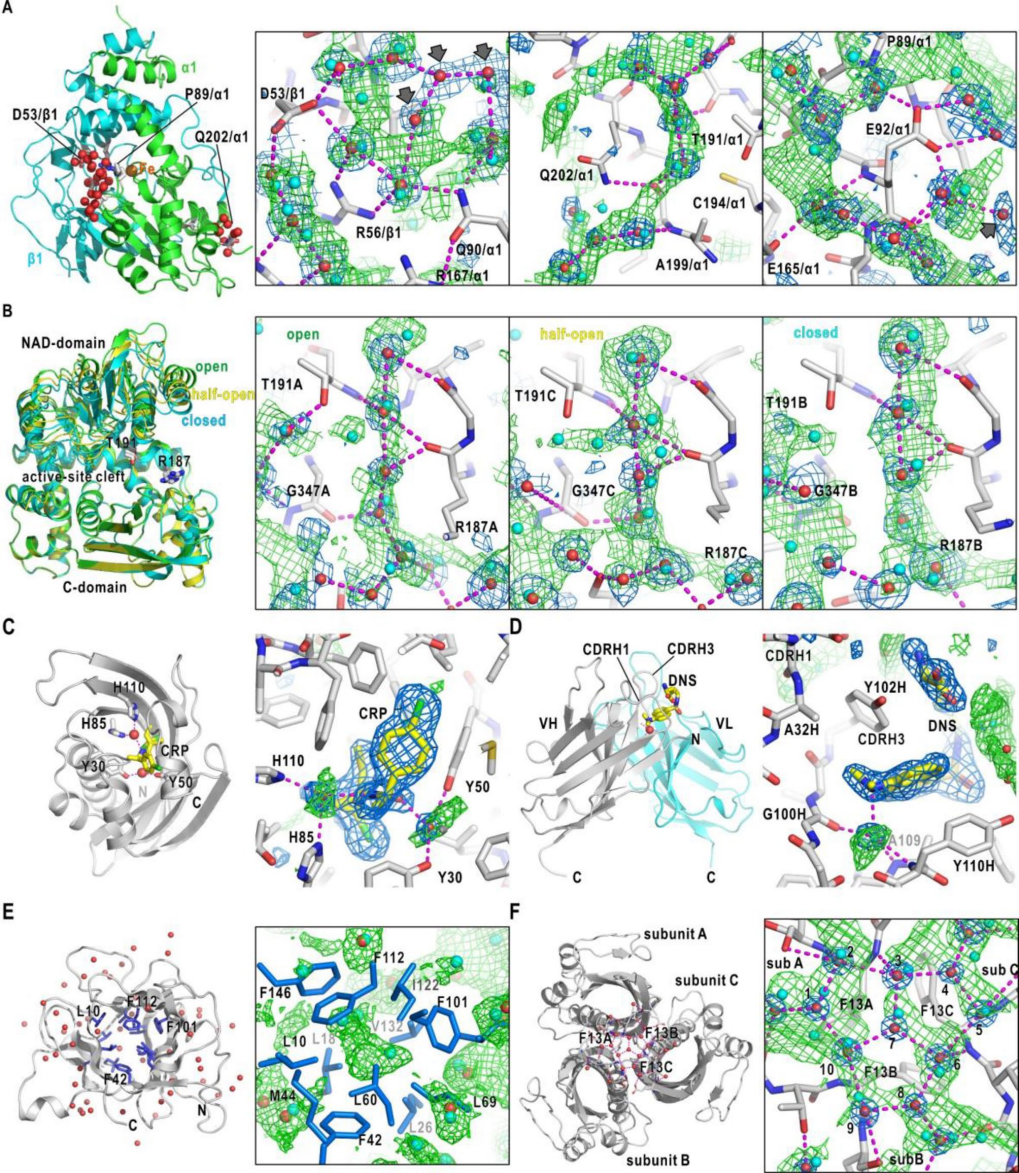
Predicted probability distributions for clusters of hydration water molecules in the first-layer class. (**A**) Prediction for three clusters of hydration water molecules of NHase. (**B**) Prediction of columnar arrangement of hydration water molecules in the open, half-open, and closed states in the NAD-domain motion to open/close the active site cleft in GDH subunits. The locations of Arg187 and Thr191 are shown in the left panel. (**C**) Whole structure of SDH subunit in complex with its inhibitor, carpropamid (CRP) (the accession code of PDB: 2std) (left panel) and a magnified view of the inhibitor moiety in the active site pocket (right panel). (**D**) Whole structure of the anti-dansyl Fv fragment (the accession code of PDB: 1wz1) (left panel) and magnified view of the binding-site. In panels (**C**) and (**D**), blue nets are omit-difference Fourier maps of ligand molecules and hydration water molecules mediating protein–ligand interactions contoured as Fig. 2A. (**E**) Probability distribution in a hydrophobic pocket located at the center of IL-1β (the accession code of PDB: 9ilb). (**F**) Prediction of a cluster of three pentagonal arrangements on a hydrophobic surface of trimeric Phe162Ala-mutated SDH (the accession code of PDB: 1idp).

As examples of hydration prediction for surfaces of a protein in a stationary conformation, Fig. 4A compares the NN-predicted probability distributions with the locations of hydration water molecules on the three surface regions of NHase. On the surface formed by the side chains of Asp53/β1, Arg56/β1, and Gln90/α1, eight water molecules in the first-layer class and three without direct interactions with the protein atoms were identified in the crystal structure. The probability distribution covered almost all the water molecules in the first-layer class, and the local maxima were located within 0.4–1.3 Å from the hydration sites. In contrast, low probability distributions appeared near two of the three crystal-water molecules without direct contact with the protein atoms as indicated by the gray arrows in Fig. 4A.

On the surface around the Gln202/α1 side chain, the NN yielded an arc-shaped probability distribution covering the seven crystal-water molecules, and the local maxima were located in the distance range of 0.4–1.7 Å from the molecules. In addition, the predicted distribution suggests the presence of two additional hydration sites covering the peptide bond of Gln202/α1-Val203/α1, which were missing in the crystal structure. Around the Glu92/α1 side chain, the predicted probability distribution overlapped with ten hydration water molecules, and the local maxima were located in the 0.2–1.3 Å range from the water molecules. Water molecules with thermal factors greater than 30 Å^2^ tended to be located at the edge of the 80% contoured probability distribution.

Next, we examined whether the NN reproduced the hydration structures of metastable conformations appearing in the internal motions of proteins. Unliganded GDH undergoes spontaneous hinge-bending motions of the NAD-binding domain to open/close the active-site cleft situated between the NAD-binding and C-domains^5,7^ (Fig. 4B). In a crystal, each subunit is trapped in one of three metastable conformations, i.e., open (subunit A), half-open (subunits C, D, and F), and closed (subunits B and E) in the domain motion, and the hydration structure changes are visualized for the three conformations^22^ (Fig. 4B). Changes in the columnar arrangements of hydration structures surrounding Arg187, Thr191, and Gly347, which form an edge region of the active-site cleft, are key factors in the regulation of NAD-domain motion. The predicted probability distributions almost reproduced the columnar arrangement of hydration water molecules in the three metastable conformations, with the maxima located in the distance range of 0.1–1.5 Å from the identified hydration water molecules, suggesting that the NN could predict the hydration structures of proteins in metastable states in global conformational changes.

Based on these examples, we conclude that the NN is capable for predicting the probability distributions of hydration water molecules in the first-layer class on hydrophilic protein surfaces, and that the NN can predict hydration structures occurring in concert with the conformational transition of proteins.

### Prediction for liganded active-sites

As hydration water molecules mediate molecular interactions between proteins and ligand molecules, the prediction of hydration sites is necessary for understanding the association mechanisms of ligand molecules^37,38^. Here, we show preliminary tests regarding the feasibility of the NN to predict hydration sites mediating protein-ligand interactions.

In scytalone dehydratase (SDH) inhibited by a tight-binding inhibitor, carpropamid ((1RS,3SR)-2,2-dichloro-N-[(R)-1-(4-chlorophenyl)ethyl]-1-ethyl-3-methylcyclopropanecarboxamide)^39^, two hydration water molecules contribute to fix the central part of the inhibitor molecule at the bottom of the active-site pocket. One water molecule forms hydrogen bonds with the amide group of the inhibitor molecule and two histidine side chains, and another forms hydrogen bonds with the tips of two tyrosine side chains and fixes the carbonyl group of the inhibitor molecule. The NN-predicted hydration probability distribution overlapped with the two hydration water molecules.

Fv part of immunoglobulin G (IgG), which plays a key role in immunological responses, recognizes antigen/hapten molecules together with hydration water molecules. As an example, Figure 4D shows the structure of a Fv fragment of anti-dansyl IgG in complex with dansyl-lysine^40^. One hydration water molecule, which hydrated a pocket formed by the complementary determining regions (CDRs) H1 and H3, contributes to recognize the middle of the hapten. The NN-predicted hydration site overlapped with the hydration water molecule.

### Prediction for hydrophobic cavity and surface

Hydration water molecules in hydrophobic cavities and on hydrophobic surfaces are predominantly in isotropic van der Waals contact with non-polar atoms, and the arrangement of hydration water molecules is influenced by the locations of hydration water molecules forming hydrogen bonds with polar protein atoms^6,8^. In addition, hydration water molecules with positional disorder on hydrophobic surfaces are difficult to identify by X-ray crystallography. Therefore, in contrast to hydration patterns on hydrophilic surfaces, regular arrangements of hydration water molecules are rarely identified on hydrophobic surfaces^41^, except for pentagonal arrangements^8,42^. To date, it has been very difficult to predict the hydrophobic hydration of proteins using knowledge-based approaches because of the lack of regular arrangements of hydration water molecules around hydrophobic surfaces. However, because the dataset included the distribution patterns of hydration water molecules near non-polar groups, the NN may be applicable for the prediction of hydration probability on hydrophobic surfaces and cavities. Here, we show representative examples of NN prediction for a hydrophobic cavity in interleukin-1β (IL-1β)^43^ and the hydrophobic surface of unliganded Phe162Ala-mutated scytalone dehydratase (SDH)^44^.

In the core of IL-1β, a cavity of approximately 7 × 8 × 8 Å^3^ is formed by 12 hydrophobic residues^45^ (Fig. 4C). A nuclear magnetic resonance study pointed out the presence of hydration water molecules in a disordered arrangement in the core^45^. The NN predicted a hydration distribution with a significant probability larger than 80%, in which two or three hydration water molecules could occupy and overlap the electron density maps of disordered hydration water molecules in X-ray crystallography^46^.

A pentagonal arrangement of hydration water molecules, which is a typical hydration pattern in clathrate structures to hydrate gas molecules^47^, are present on protein surfaces^6,8,42^. Figure 4D shows an NN-predicted probability distribution on the hydrophobic surface of Phe162Ala-mutated SDH (SI Appendix, Table S2), where three pentamers composed of ten hydration water molecules hydrated three phenylalanine side-chains. The probability distribution covered six hydration sites at the 80% level and all sites at the 70% level. The local peaks of the probability distribution were located in the distance range of 0.4–1.1 Å from hydration water molecules 1, 2, 3, 5, 6, 7, 9, and 10. In particular, the probability maximum appeared within 0.9 Å from hydration water molecule 7, which contacted three phenylalanine side chains alone. The predicted distribution lacked three-fold rotational symmetry, probably because of the incomplete symmetry among the subunits and the sensitivity of the NN-predicted distribution to small differences in atom positions.

## Discussion

**Figure 5:**
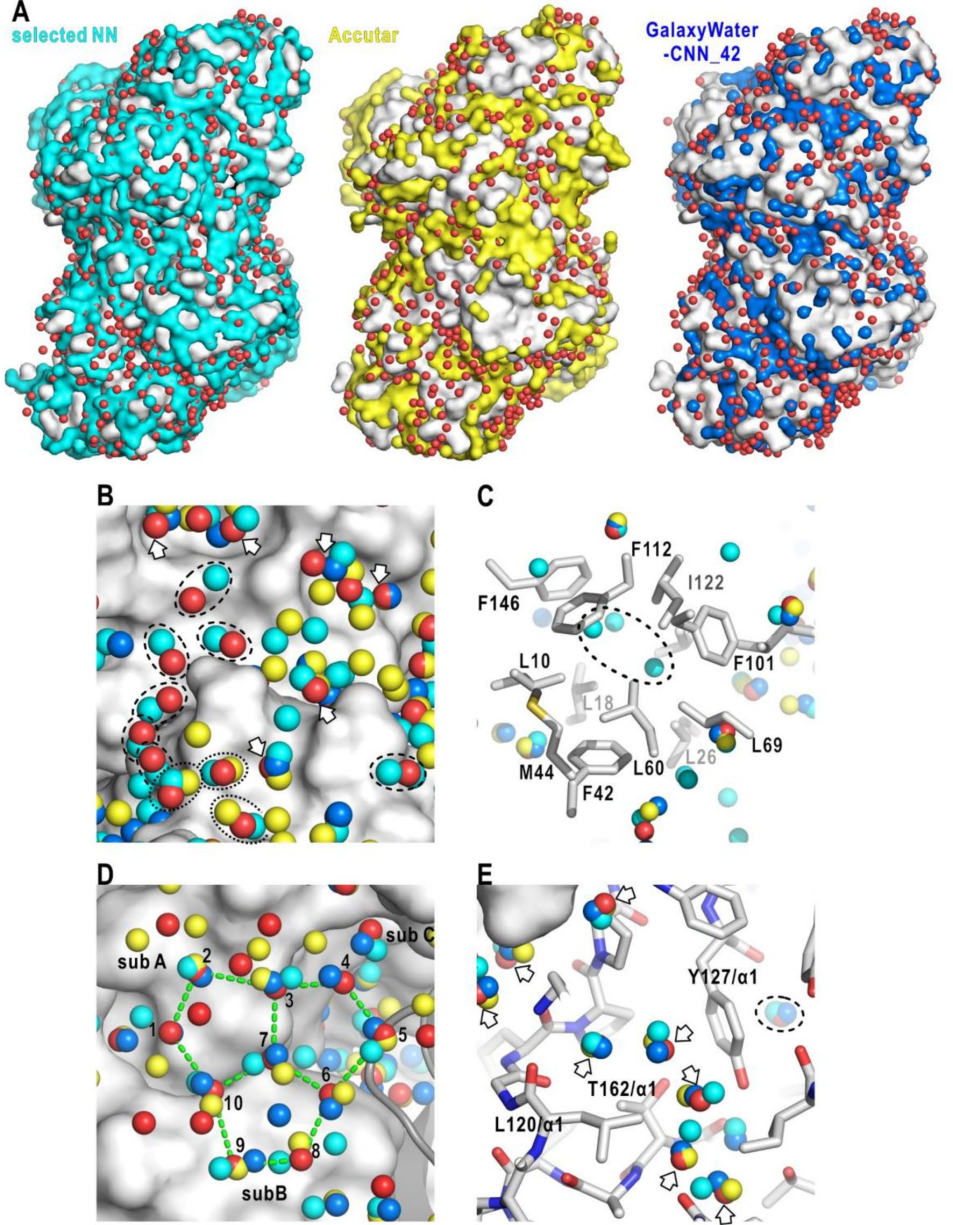
Comparison of the predicted hydration sites among our NN and the two other NN-based methods. (**A**) Distributions of the crystal-water sites in the first-layer class (red spheres) and the predicted hydration sites from our NN (cyan surface in the left panel), Accutar^30^ (yellow surface in the middle panel) and GalaxyWater-CNN_42^31^ (blue surface in the right panel) on the surface of NHase. (**B**) Magnified view of the distributions of crystal-water sites in the first-layer class and the predicted hydration sites on NHase. In the following panels, the spheres indicating the hydration sites as colored in panel (**A**). The arrows indicate the crystal-water sites predicted by the three NN-based methods. Dotted and dashed circles indicate crystal-water sites predicted by our NN and Accutar and by our NN only, respectively. (**C**) Distributions of predicted hydration sites in the hydrophobic core of IL1-β as illustrated in Fig. 4C. The dashed line indicates the hydrophobic-hydration area. (**D**) Distribution of crystal-water and predicted sites on the hydrophobic surface of Phe162Ala-mutated SDH as illustrated in Fig. 4D. (**E**) Crystal-water of the inside class and predicted hydration sites in the interior of NHase.

Here, we compare the performance of our NN with that of two other NN-based hydration prediction methods, GalaxyWater-CNN_42^30^ and Accutar^31^, with respect to the distributions of predicted hydration sites (Fig. 5 and SI Appendix, Fig. S3), *MAD* and *RMSD* (Table 1 and SI Appendix, Table S3). As an example, Fig. 5A compares the distributions of crystal-water sites of NHase and the predicted sites in the first-layer class by the three NN-based methods. Our NN predicted hydration sites uniformly cover the NHase surface, while sites predicted by the other methods were frequently absent from the surface bulges and crowded mainly on grooves. As a result, the surface coverage of the predicted hydration sites by our NN was 75%, and those by the Acctar and GalaxyWater-CNN_42 were 58% and 58%, respectively. This tendency was also observed in protein structures used in the validation test of GalaxyWater-CNN^30^. Hydration sites predicted by our NN covered 73–75%, and those by the other two were in the range of 40–50% (SI Appendix, Table S3 and Fig. S3).

Figure 5B compares the three methods regarding the reproducibility of the crystal-water sites of the first-layer class. The three methods almost reproduced several crystal-water sites indicated by arrows. On the other hand, at crystal-water sites indicated by dotted and dashed circles, one or two methods failed to predict, although the sites were free from crystal contacts. The three methods were also compared with respect to the prediction of hydrophobic regions. In the hydrophobic core of IL-1β (Fig. 5C), the hydration sites expected from experimental data^45,46^ were predicted by our NN only. In contrast, on the hydrophobic surface of the Phe162Ala-mutated SDH, all the three methods predicted hydration sites near the crystal-water sites in the clathrate arrangement (Fig. 5D).

Regarding the *MAD* and *RMSD* for the crystal-water sites in the first-layer class of GDH and NHase, our NN yielded the best scores (Table 1). In the two other methods, many sites were distant from the crystal-water sites, while some predicted sites almost overlapped with the crystal-water sites as seen in Fig. 5B–D. For the inside class, the three methods reproduced almost all the crystal water sites (Fig. 5E). Particularly, the two other methods frequently predicted hydration sites almost overlapping with the crystal-water sites and yielded the *MAD* and *RMSD* scores better than those of our NN (Table 1), probably because the two methods were trained by the datasets explicitly including the stereochemical information on hydration sites. We also compared the scores for nine small protein molecules used in the validation test of GalaxyWater-CNN^30^ (SI Appendix, Table S3). The two scores of our NN were better than those of the other two methods.

Therefore, our NN method showed somewhat better performance than the other two NN-based methods with respect to the distributions of hydration sites over protein surfaces and in the two scores for assessing positional differences between predicted and observed sites except for the inside class. Since the performance of an NN-based method likely depends on the NN architecture, the type and the size of the training datasets, the comparison suggested that better results may be obtained by NNs trained by both the distribution of protein atoms around the crystal-water sites and the information on stereochemistry and interaction energies at the sites.

The constructed NN displayed the average accuracy and loss scores of 0.873 and 0.298, respectively, and showed the performance as demonstrated in Figs. 3, 4 and 5, Table 1, SI Appendix Fig. S3 and Table S3. Based on the correlation between the accuracy/loss scores and the predicted results, although the performance of NN-based prediction method depends on the size of the database, the architecture of NN and so on, the accuracy and loss scores may be a good measure necessary to achieve, at least, the performance like our NN and to develop NN-based methods with better performance than our present NN.

Besides the NN-based hydration prediction methods, we compared the performance of our NN with those of knowledge-based and MD-based prediction methods. The hydration distributions predicted by our NN completely covered the probability distribution calculated using the empirically determined hydration distributions around polar atoms^21,22^, and overlapped with the solvent densities^25,26^ deduced from a MD simulation with an appropriate force field and water model^6,8,27^.

The NN was optimized using the dataset composed of ‘water-present’ and ‘water-absent’ atom groups. Water-present groups specified by crystal-water sites and contributed to increasing the hydration probabilities around atom groups in similar arrangements to water-present groups. However, the probabilities will decrease around atom groups in a similar arrangement to water-absent groups.

In the dataset, two types of water-absent groups were present. One included a set of atom groups that were never hydrated. The other was a set of atom groups probably hydrated but lacking their hydration water molecules in crystal structures owing to the positional disorder. Therefore, atom groups in the latter type were incorrectly classified into the water-absent group, and the incorrect assignment may bias the reduction in hydration probability around atom groups arranged similar to the latter type.

Although the results shown in Figs. 2, 3 and 4 suggested that the influence of the bias described above was minor, a more rigorous assignment of water-absent groups is required. One method is the two-step optimization of the NN. The dataset for the first optimization is the same as that described in the Methods section. In this preliminary optimization, although the parameters in the CB will be optimized to adequately predict the hydration distributions, those in the FCB have room for further refinement. In the second optimization stage, the parameters in the FCB are refined using the training dataset, which is prepared from crystal structure models solved at a resolution beyond 1 Å by focusing on hydration water molecules with B-factors smaller than 30 Å^2^. Moreover, in the second stage, the FCB is newly constructed as a regression model for water-present probabilities based on the B-factors of water molecules, so that the penalty would be adequately suppressed for the potential location of the hydration water molecules.

In the ideal prediction of hydration structures based on crystal structures, the predicted hydration sites coincide with crystal-water sites. The NN was optimized using the dataset for the distribution patterns of protein atoms, but not for the stereochemical geometry of hydrogen bonds, because the dataset may implicitly contain the stereochemical characteristics of atomic contacts. For the inside class, most of the local maxima in the predicted hydration probability distributions were located within 0.7 Å from the experimental observations, probably owing to several interactions in the cavities (Table 1 and Fig. 3). Regarding the first layer class, local maxima in the predicted distribution were at times greater than 1.3 Å, i.e., half of the typical hydrogen-bond distance, from the crystal-water sites (Table 1 and Fig. 4). Because the local maxima in the predicted probability distributions did not always suggest appropriate hydration sites, the protocol has room for improvement to minimize the positional differences in hydration sites between the prediction and experiment. The following two strategies may improve the prediction of potential hydration sites.

One is the combinational use of the NN-predicted hydration probability distribution and the directionality of hydrogen bonds reported by the database analysis^21^ and MD simulation^27^. In addition, the frequency distributions of hydration water molecules are available for the rotatable O–H groups in the serine and threonine side chains^27^. The second is the introduction of an input channel regarding the distribution patterns of hydrogen atoms in amino acid residues. Owing to progress in synchrotron X-ray crystallography^48^ and neutron crystallography^49^, a number of hydrogen atoms have been identified in proteins. In addition, the positions of the hydrogen atoms are virtually generated by protocols using crystallographic refinement^50^ and MD simulations (https://manual.gromacs.org/documentation/current/onlinehelp/gmx-pdb2gmx.html).

As the constructed NN predicts the hydration over hydrophobic surfaces of transmembrane regions, it is necessary to exclude the prediction, for instance, by multiplying the knowledge-based prediction on the hydration structures of hydrophilic surfaces^8,22^. In addition, two preliminary tests in Fig. 4C,D suggested the capability of the NN to predict hydration sites in protein-ligand interface. However, the further training is necessary for protein complexed with various types of ligand molecules for understanding the association mechanisms and designing inhibitor molecules^37,38^.

## Methods

### Preparation of datasets

The datasets for training the NNs were prepared from crystal structure models of proteins available from the Protein Data Bank^51^. To collect a number of hydration patterns on various surface types, we selected proteins with molecular weights greater than 100 k. Furthermore, for an unambiguous collection of hydration patterns, we selected 2145 crystal structures (SI Appendix, S1), which were solved at a resolution of 1.6‒1.8 Å using diffraction data collected at cryogenic temperature, and displayed crystallographic *R*-factors and *R*_free_-factors smaller than 0.20 and 0.25, respectively. By using the PISCES site (http://dunbrack.fccc.edu/pisces/PISCES.php)^52^, the 2145 protein structures were separated into 1,066 groups under the sequence similarity threshold of 30%. This implied that two protein molecules had a sequence similarity on an average.

As reported previously, each local hydration structure predominantly depends on the interactions between a hydration water molecule and protein atom groups and satisfies the tetrahedral hydrogen-bond geometry of water molecule^8,21^. Therefore, the NN was optimized with respect to the local hydration structures. To collect the distribution patterns of protein atoms surrounding hydration water molecules, we used a trimming box composed of 0.25 × 0.25 × 0.25 Å^3^ voxels. The size of the trimming box was set to 41 × 41 × 41 voxels, corresponding to 10.25 × 10.25 × 10.25 Å^3^ (Fig. 1A) based on the frequency distributions for water-water and water-protein atom distances (SI Appendix, S2 and Fig. S1). For each protein structure model, surfaces and cavities with a non-zero ASA^53^ were randomly scanned using the trimming box.

When a hydration water molecule is present in the trimming box, the center of the box is placed at the water molecule. The distribution of the protein atoms was designated as the ‘water-present’ pattern. In addition, the distribution of protein atoms without hydration water molecules was collected as a ‘water-absent’ pattern to equalize the number ratio between the water-present and water-absent patterns in the subsequent training of NNs. Each distribution pattern was voxelized separately with respect to the atomic species, i.e., the carbon, nitrogen, oxygen, and sulfur atoms (Fig. 1A). Finally, a dataset composed of 5,310,762 patterns (hydration sites) was obtained from 2145 protein structures. Of these patterns, 70% were randomly selected as the training dataset and 30% were used as the validation dataset. This selection was independent from protein structures and sequences because of the characteristic of the local hydration structures as described in the Results section.

In addition, for the extra validation of the selected NN, we prepared a set of 300 crystal structures of proteins with the molecular weights of 50–100 k. The structures were taken from a cluster of protein structures displaying less than 30% sequence identity^51^. The resolution of each crystal structures was in the range of 1.0‒1.8 Å. The test dataset yielded 174,796 patterns of hydration sties.

To efficiently calculate the hydration probabilities, coarse datasets were prepared independently from the datasets described above, using a trimming box composed of 21 × 21 × 21 voxels of 0.50 × 0.50 × 0.50 Å^3^. The NNs optimized using the coarse datasets predicted the preliminary hydration distributions to roughly indicate the potential hydration sites.

### Architecture of constructed neural network

We constructed NNs based on the three-dimensional convolutional neural network^54^. Each NN was composed of a CB and an FCB, classifying the data provided by the CB. (Fig. 1B).

In the CB, the given distribution patterns of protein atoms were processed by convolution units (CU), each comprising two convolution layers (CL) followed by a pooling layer (PL) to down-sample the CL output and a dropout layer to avoid over-learning^55^. A rectified linear function^56^ was applied to the output of each layer as an activation function throughout the NN.

The first CL had four channels to independently process the distribution patterns of the four atomic species (carbon, nitrogen, oxygen, and sulfur atoms). A three-dimensional convolution filter (3D-CF) was applied to each distribution pattern of atoms to extract representative quantities as follows:

$$u_{ijk} = \sum\limits_{p = 1}^{F} {\sum\limits_{q = 1}^{F} {\sum\limits_{r = 1}^{F} {x_{i + p,j + q,k + r} \, f_{pqr} ,} } }$$

where $x_{i + p,j + q,k + r}$, $f_{pqr}$, and $u_{ijk}$ are the input data, convolution filter with a size of $F \times F \times F$, and output data, respectively. In the pooling layer, we used the max-pooling^57^ with a pooling size of $P \times P \times P$. The data input to the layer was subsequently downsampled.

The results from the FCB composed of several nodes were evaluated using the loss function of binary cross-entropy. For the output $y \in \left[ {1,0} \right]$ for the positive class (*d* = 1) or negative class (*d* = 0), the loss function is calculated as:

$$L\left( {\mathbf{w}} \right) = - \left\{ {d\log y + \left( {1 - d} \right)\log \left( {1 - y} \right)} \right\},$$

where ${\mathbf{w}}$ represents all the parameters of the model (Fig. 1B). Through training, the 3D-CFs in the CLs were optimized to minimize the loss function.

We constructed six NNs composed of different numbers of CBs, channels in CLs, nodes, and layers in FCB (SI Appendix, S3 and Table S1), as well as the size of the 3D-CF. The NNs were optimized using the adaptive moment estimation^58^. When the NN optimized using the fine dataset was directly applied to proteins with molecular weights exceeding 100 k, large computation times were required to obtain the hydration probability distributions. To reduce the computation time, we created two NNs with the same architecture: one NN was optimized using the coarse dataset (designated NN-coarse), and the other using a fine dataset (NN-fine). We selected the NN that efficiently yielded the hydration probability (see the Results section).

Among the NNs, we selected a set of NN-coarse and NN-fine by inspecting the accuracy and loss scores for the validation and test datasets (SI Appendix, S3 and Table S1), as well as by assessing the frequency distribution of hydration probability at the sites of hydration water molecules found in the crystal structures (SI Appendix, Fig. S2). For evaluating the selected NN, we used the following validation measure defined as

$$\begin{array}{*{20}l} {accuracy = \frac{TP + TN}{{TP + FP + TN + FN}}} \hfill \\ {precision = \frac{TP}{{TP + FP}}} \hfill \\ {recall = \frac{TP}{{TP + FN}}} \hfill \\ {F - measure = \frac{2 \times precision \times recall}{{precision + recall}}} \hfill \\ \end{array} ,$$

where $TP$ and $FP$ are true positive and false positive. $TN$ and $FN$ are the true negative and false negative, respectively. The *accuracy* metric reports the rate of correctness to the total test data. The *precision* is the positive predictive value, which indicates the rate of correctness to the predicted positives. The *recall* is the true positive rate, which indicates the rate of correctness to the positive label data. The *F-measure* is the harmonic mean of precision and recall. The values of the three measures in the hydration prediction for the set of 300 protein structures are listed in Table 1.

### Hydration probability

In the first step, to calculate the hydration probability over the surface of a targeted protein, surfaces and cavities with non-zero ASA were scanned using a box composed of 21 × 21 × 21 voxels of 0.50 × 0.50 × 0.50 Å^3^. Next, the coarse-NN yielded hydration probability distributions at a resolution of 0.50 × 0.50 × 0.50 Å^3^ voxels by inspecting the distribution pattern of protein atoms around the center voxel. This procedure was iteratively performed for all voxels that were located within 4 Å from protein atoms with non-zero ASA values and helped to identify candidates for surfaces to be hydrated.

In the second step, the fine-NN was applied to each candidate suggested in the first step, and yielded the hydration probability at a resolution of 0.25 × 0.25 × 0.25 Å^3^ voxels using the same calculations as in the first step. The final hydration probability output was obtained in the MRC format^59^. When the probabilities were greater than an appropriate threshold, the local maxima were selected from the probability distributions as predicted hydration sites.

### Prediction scores

We evaluated the manner in which the local maxima of the probability distribution approximated the nearest hydration sites using the mean absolute positional deviation (*MAD*) and root-mean-square deviation (*RMSD*) scores, defined as follows:

$$MAD = \frac{1}{N}\sum\limits_{i = 1}^{N} {\left| {{\mathbf{r}}_{i}^{c} - {\mathbf{r}}_{i}^{p} } \right|} , \, RMSD = \sqrt {{{\sum\limits_{i = 1}^{N} {\left( {{\mathbf{r}}_{i}^{c} - {\mathbf{r}}_{i}^{p} } \right)^{2} } } \mathord{\left/ {\vphantom {{\sum\limits_{i = 1}^{N} {\left( {{\mathbf{r}}_{i}^{c} - {\mathbf{r}}_{i}^{p} } \right)^{2} } } N}} \right. \kern-0pt} N}} ,$$

where ${\mathbf{r}}_{i}^{c}$ and ${\mathbf{r}}_{i}^{p}$ are the positions of an experimentally identified hydration site and the local maximum of probability distribution near the site, respectively. $N$ is the number of hydration sites targeted in the evaluation.

### Coverage of protein surface area by predicted hydration sites

To compare the performance of NN-based prediction methods, we used surface coverage, the area ratio of predicted sites to a whole protein surface. The protein surface area covered by a predicted site was set to 20 Å^2^, which was estimated for a single hydration water molecule in the first-layer class by a systematic analysis of experimental data^6,8^. The surface area was calculated by PyMOL^60^.

### Coding and computation

The NNs were developed using the Python language with some routines obtained from TensorFlow (Google Brain, USA). All computations were performed on a high-speed computer server composed of two Intel Xeon Gold 6226R (16 cores, 16 threads) (HPCT W216gs-DL, HPC Tech, Japan) equipped with a GPU card (NVIDIA Quadro RTX 8000 of CUDA version 10, NVIDIA, USA). The computational times of the selected NN-fine on the server are listed in Table SI4 for proteins with different molecular weights. Therefore, in comparison with other NN-based methods^30–32^ and 3D-RISM^28,29^, the computational cost of our NN based on the simple CNN architecture is low enough to predict hydration structures over multi-domain protein structures, such as GDH comprising 2514 residues, even using the computer server.

## Supplementary Information

Supplementary Information.

## Data Availability

The datasets used and/or analyzed during the current study available from the corresponding author on reasonable request.
