# Supplementary material for: Prediction of hydrophilic and hydrophobic hydration structure of protein by neural network optimized using experimental data
Source: Sci Rep. 2023 Feb 7;13:2183. doi: 10.1038/s41598-023-29442-x (PMC9905073; doi:10.1038/s41598-023-29442-x)
Supplement: Supplementary file 1 — Supplementary Information. [file 41598_2023_29442_MOESM1_ESM.docx]

**Supplementary Information**

Prediction of Hydrophilic and hydrophobic hydration structure of protein by neural network optimized using experimental data

Kochi Sato^1,2^, Mao Oide^1,2^ and Masayoshi Nakasako^1,2,*^

^1^Department of Physics, Faculty of Science and Technology, Keio University, 3-14-1 Hiyoshi, Kohoku-ku, Yokohama, Kanagawa 223-8522, Japan.

^2^RIKEN SPring-8 Center, 1-1-1 Kouto, Sayo-cho, Sayo-gun, Hyogo 679-5148, Japan

^3^PRESTO, Japan Science and Technology Agency, Chiyoda-ku, Tokyo, 102-0076, Japan

^*^ To whom correspondence should be addressed. Phone: +81-45-566-1713. Fax: +81-45-566-1672.

E-mail: nakasako@phys.keio.ac.jp.

**S1. List of the Protein Data Bank accession codes of 2,145 crystal structures used for preparing the dataset used in the training constructed neural networks.**

1a12, 1aom, 1aoq, 1as6, 1bav, 1bu7, 1ccw, 1cyd, 1d0i, 1d8w, 1dgg, 1dp0, 1dps, 1duv, 1dwk, 1e3d, 1e6p, 1e6y, 1eef, 2rfw, 2rjt, 2uxq, 2uz0, 2uz1, 2v2g, 2v61, 2v63, 2vae, 2vb7, 2vbf, 2vbg, 2vck, 2vjq, 2vk1, 2vr4, 2vtf, 2vvh, 2w08, 6pt9, 6ptk, 6pud, 6pug, 6q2c, 6q9c, 6qcl, 6qjc, 6qne, 6qng, 6qo0, 6qpv, 6qpx, 6qpz, 6qsi, 6qya, 6qyi, 6qz4, 6r29, 6r2a, 6s49, 6s4d, 6s4g, 6s65, 6s6g, 6s7s, 6s87, 6s8v, 6scg, 6sj0, 6slf, 6smz, 6t0f, 6t5q, 6t6r, 6ta2, 6tac, 6tbk, 6tld, 1eex, 1m2w, 1oja, 1tr0, 1y2m, 2ad8, 2cdb, 2f5x, 2ib5, 2jer, 2p2o, 2pwf, 2rfq, 2w3x, 2xh2, 2yky, 2ztl, 3bn1, 3dds, 3ehw, 3gn6, 2q28, 2q2a, 2q62, 2q7q, 2qee, 2qin, 2qjj, 2qkf, 2qm6, 2qub, 2qul, 2quy, 2r2d, 2r5n, 2r8p, 2rcm, 2rcu, 2rdz, 2pj1, 2pj3, 2pj5, 2pj7, 2pj8, 2pja, 2pjb, 2pjc, 2pn8, 2poc, 2pp7, 2pp9, 2ppa, 2ppd, 2ppe, 2ppf, 2ps2, 2pwd, 6r6u, 6r88, 6r9w, 6rim, 6rk7, 6rke, 6rkp, 6rkz, 6rl0, 6rpc, 6rs0, 6rtt, 6rud, 6rue, 6rxd, 6rxg, 6rxq, 6rxr, 6s0o, 6s18, 5on5, 5on8, 5on9, 5opq, 5oss, 5ovq, 5oya, 5rl9, 5rub, 5rvw, 5rvx, 5rvy, 5rw0, 5sx1, 5sxr, 5sxw, 5sxx, 5syh, 5syi, 5syk, 2jis, 2jke, 2nt0, 2o2p, 2o4m, 2o70, 2o8j, 2ogr, 2ohh, 2ojy, 2oov, 2oqe, 2osw, 2otu, 2oui, 2ow7, 2ox4, 2oyl, 2ie2, 2ifc, 2igk, 2ign, 2ij5, 2iov, 2ipi, 2iup, 2iwk, 2iy6, 2j1m, 2j5i, 2j5n, 2j78, 2j91, 2jah, 2jbf, 2je8, 2aen, 2agz, 2apj, 2bbk, 2bek, 2bhp, 2bo9, 2bt4, 2byw, 2c31, 2c42, 2c5q, 2c65, 2c67, 2c6q, 2c75, 2c76, 2cb2, 2cch, 2fdv, 2fgz, 2fh6, 2fym, 2g50, 2gai, 2gbw, 2gfq, 2gmy, 2gn0, 2gx2, 2h88, 2hj4, 2hpo, 2hrd, 2hzk, 2i62, 2i8a, 6to1, 6tq5, 6tzj, 6tzl, 6tzu, 6u0y, 6u1v, 6ub2, 6ufp, 6uuv, 6uwe, 6uxt, 6uya, 6uyh, 6uzt, 6v1o, 6v25, 6v26, 6v43, 6y1w, 6y41, 6y73, 6y9e, 6yhh, 6yt2, 6z7r, 6z9g, 6zhk, 6zjq, 6ztv, 6zxu, 7c0d, 7c3h, 7c3l, 7coi, 7k2x, 7k73, 7kcd, 7kq6, 2cdu, 2cfc, 2cfd, 2cvp, 2cvz, 2cxo, 2cxp, 2cxr, 2cxu, 2d1w, 2d1y, 2d3z, 2dbw, 2dbx, 2de6, 2deb, 2dg1, 2dg5, 2dvm, 2dvt, 2dvx, 2e11, 2e1v, 2ea7, 2eb5, 2eb6, 2ehu, 2eit, 2ej0, 2ekq, 2epn, 2ez9, 1tt0, 1tw9, 1twy, 1u0f, 1u0g, 1u60, 1u8f, 1u8v, 1uc4, 1uqr, 1uwl, 1uxl, 1v02, 1v7z, 1vdk, 1vgg, 1vkn, 1vl2, 1vlp, 1vph, 1vr5, 1w27, 1w2w, 1w3i, 1w4n, 1w96, 1wap, 1x0c, 1x6v, 1x74, 1xa1, 1xk4, 1xmp, 1xrt, 1xs1, 1xu7, 1xx1, 1y7b, 1yqp, 1yve, 1z02, 1z03, 1z0s, 1z7a, 1zdq, 1zja, 1zjb, 1zlq, 1zmt, 1zo9, 1zoa, 1zxi, 1zz0, 1zz3, 2a9e, 1ooy, 1oq1, 1p0z, 1p1j, 1p3d, 1p7g, 1p80, 1pam, 1pn0, 1px3, 1px4, 1pzg, 1q52, 1q74, 1q8f, 1qbi, 1qmg, 1qwl, 1qwm, 1r1n, 1r33, 1r7a, 1rjd, 1rm6, 1rx0, 1ryi, 1s3e, 1s5u, 1sio, 1sw5, 1sy7, 1t0b, 1t0t, 1t3q, 1t6g, 1t7q, 1tqj, 2w93, 2wan, 2wc4, 2wnq, 2wtm, 2wvg, 2wvv, 2wy3, 2wya, 2x1d, 2x3h, 2x4y, 2x8r, 2x8t, 2xc1, 2xf2, 2xfn, 2xfp, 2xfw, 2xh0, 3itl, 3itv, 3itx, 3ixq, 3jq6, 3jq7, 3jqc, 3jqd, 3jqf, 3ju5, 3jxg, 3jze, 3k40, 3k4c, 3k4l, 3kd2, 3kgd, 3kl0, 3klk, 3kmv, 3gp9, 3gza, 3gzd, 3h09, 3h46, 3h4h, 3h71, 3h72, 3h73, 3hlh, 3hps, 3hq1, 3i4z, 3ia2, 3ihv, 3ii9, 3ilw, 3inj, 3iqe, 3bof, 3bv6, 3bve, 3bvl, 3bza, 3c3j, 3c61, 3c7t, 3c8w, 3cff, 3cli, 3cmc, 3cq5, 3d01, 3d1i, 3d50, 3db2, 3ddg, 3ddq, 2xh9, 2xi2, 2xi3, 2xii, 2y08, 2y0c, 2y0e, 2y27, 2y3r, 2y3s, 2y51, 2y52, 2y7f, 2y8n, 2ya4, 2ya8, 2yav, 2yax, 2yhi, 3zx3, 4a1i, 4a6t, 4a6u, 4a7a, 4adi, 4adm, 4ag3, 4agi, 4aia, 4aj2, 4ajj, 4ajl, 4ak4, 4al4, 4ao5, 4ax3, 4b12, 4b2n, 4b2o, 3mi5, 3ml1, 3mm5, 3mn1, 3mof, 3msj, 3mvk, 3mvr, 3mvw, 3mvx, 3mvz, 3mwp, 3myv, 3mz9, 3mzb, 3n2b, 3n2n, 3n33, 3n5i, 3n5w, 3n65, 3n66, 3n6q, 3n81, 3n9r, 3na5, 3ndv, 3nez, 3ngj, 3no6, 3nrr, 3nrz, 3nug, 3nvw, 3o0d, 3o0k, 3o0q, 3o0y, 3o1w, 3o4w, 3wia, 3wih, 3wiu, 3wj2, 3wlv, 3wol, 3won, 3woo, 3wpu, 3wrz, 3ws1, 3wsp, 3wu4, 3wu6, 3x0u, 3zeu, 3zgo, 3zli, 3znu, 3oa5, 3oa8, 3oam, 3occ, 3oid, 3ois, 3ojc, 3ojj, 3ojk, 3ojn, 3ojt, 3oks, 3on6, 3oow, 3or1, 3os7, 3ot9, 3otw, 3ow1, 3owm, 2ymm, 2ymu, 2ync, 2yne, 2yoj, 2yoo, 2yqu, 2yr4, 2yw3, 2z1o, 2z6z, 2z8k, 2zad, 2zbl, 2zbt, 2zct, 2zo5, 2zog, 2zon, 3p24, 3p2c, 3p4g, 3p9a, 3pde, 3pij, 3pj0, 3pk0, 3pk7, 3pn8, 3po7, 3pop, 3ppm, 3pq2, 3pq3, 3pq4, 3pq5, 3pq6, 3pq7, 3pq8, 3q14, 3q37, 3q3m, 3q43, 3q44, 3qhx, 3qit, 3qk8, 3qlj, 3qpz, 3qvq, 3qy9, 3r25, 3r3u, 3r3z, 3r5v, 3r5w, 3r8y, 3rg8, 4gji, 4glo, 4gqe, 4gt2, 4gtd, 4gvs, 4gyw, 4h18, 4h19, 4h2h, 4h31, 4h41, 4h7v, 4h8j, 4hat, 4hb2, 4hgf, 4hgg, 4hkh, 4hl7, 3kom, 3kqf, 3kru, 3krz, 3ks6, 3kse, 3kvv, 3kx5, 3kzu, 3l0q, 3l1w, 3l34, 3lcg, 3lgq, 3lim, 3lkt, 3llp, 3lm4, 3log, 4hw6, 4hze, 4i02, 4i06, 4i0w, 4i4t, 4i9d, 4i9y, 4ia6, 4icl, 4ime, 4ind, 4inh, 4ip7, 4isk, 4it9, 4itu, 4iub, 4iwk, 4iy7, 4j0c, 4j0d, 4j0h, 4j0i, 4j28, 4j4h, 4j7g, 4j7h, 4jaq, 4jaw, 4jbg, 4jch, 4jds, 4jfu, 4jhn, 4jib, 4jic, 4jl1, 4jl2, 4jtf, 4qd9, 4qfh, 4qfl, 4qhp, 4qii, 4qir, 4qjo, 4qko, 4qnk, 4qol, 4qom, 4qoo, 4qoq, 4qro, 4qs5, 4qto, 4quo, 4r1d, 4r2w, 4r84, 3knr, 3lqf, 3mi1, 3n5x, 3o5a, 3ozm, 3pr8, 3rmz, 3tk8, 3use, 3wds, 3zqo, 4b5k, 4cld, 4d7o, 4eam, 4ghf, 4hvf, 4iyo, 4jti, 4krg, 4lty, 4xeq, 4xfp, 4xgn, 4xgw, 4xhf, 4xj7, 4xjk, 4xku, 4xlo, 4xq9, 4xr4, 4xra, 4xsl, 4xz9, 4xza, 4y0h, 4y0i, 4y9j, 4ydl, 4yfb, 5fdf, 5fgb, 5fjn, 5fkq, 5fs9, 5fw0, 5fxd, 5g0a, 5g0s, 5g0u, 5g0v, 5g0w, 5g1w, 5g3p, 5g3t, 5g3w, 5g5g, 5g5y, 5gmr, 4ean, 4ear, 4eat, 4eb4, 4ego, 4enr, 4ens, 4ent, 4enu, 4env, 4eo6, 4eo8, 4eoj, 4eql, 4eqr, 4eqx, 4eqy, 4erz, 4eu5, 4eu7, 4eus, 4exl, 4f03, 4f4h, 4fb9, 4fbc, 4ffc, 4ffu, 4fn4, 4fo7, 4fuq, 4fw7, 4g2n, 4g8t, 4gh5, 4ghe, 4kri, 4krx, 4kt2, 4kvg, 4kws, 4kzp, 4l0c, 4l38, 4l3h, 4lgz, 4lh0, 4lh1, 4lh2, 4lh8, 4lhd, 4lmx, 4lnb, 4lqu, 4lts, 4lvc, 4lvd, 4lvg, 4lw2, 4lww, 4m1h, 4m1i, 4m23, 4m6p, 4mad, 4mae, 4mco, 4mdc, 4mg4, 4mhb, 4mif, 4mig, 4min, 4mj3, 4mjl, 4pip, 4plt, 4pnn, 4pnr, 4pns, 4pnt, 4po5, 4ps1, 4ptx, 4pv4, 4q0n, 4q0t, 4q17, 4q1o, 4q2u, 4q3o, 4q4u, 4q5b, 4q5c, 4b5w, 4b7f, 4b91, 4b9b, 4ba4, 4ba5, 4bc3, 4bc4, 4bfl, 4blp, 4bnw, 4bny, 4bqe, 4bz4, 4bz5, 4bz7, 4c9s, 4caf, 4cbb, 4cft, 4cle, 4clr, 4cm6, 4cmr, 4cok, 4cq1, 4cq4, 4crt, 4cx5, 4cyb, 4cyr, 4czs, 4d0s, 4d1i, 4d1j, 4d1p, 4d3b, 4d44, 4d52, 4d79, 5hwj, 5i0h, 5i1z, 5i3f, 5i4b, 5i52, 5i5d, 5i6d, 5i92, 5iau, 5ib0, 5ibx, 5ibz, 5igo, 5ih3, 5iia, 5in4, 5inr, 5inx, 4uf7, 4ufv, 4uma, 4unm, 4uop, 4upe, 4ur0, 4ur1, 4us5, 4usx, 4utt, 4uui, 4uvi, 4uzs, 4v0h, 4v3q, 4w7j, 4was, 4wbt, 5a3l, 5a4m, 5a6b, 5a8w, 5abm, 5aql, 5aqm, 5azp, 5b12, 5b1b, 5b2w, 5b3s, 5b5t, 5b7m, 5bmx, 5bon, 5bov, 5bp2, 5bph, 5bq2, 5bse, 5bvg, 5bwg, 5bxi, 5bxr, 5bxt, 5by7, 5byq, 5c05, 5c54, 5c6m, 5c8w, 5c90, 5c9i, 5cge, 5cj2, 4trm, 4tsq, 4ttg, 4ttt, 4tuh, 4tvb, 4txj, 4tyv, 4u0t, 4u3e, 4u83, 4uam, 4ubt, 4udg, 4udi, 4udk, 4udq, 4udr, 4ueq, 4d8g, 4d8y, 4d9b, 4dam, 4dm1, 4dmc, 4dnf, 4doy, 4dtw, 4dub, 4due, 4duf, 4dws, 4dxi, 4dz4, 4dzi, 4e1o, 4e6m, 4e8d, 4e9v, 5iol, 5iq0, 5iq1, 5iq2, 5iq3, 5irf, 5iwg, 5ix0, 5ixe, 5iyz, 5j72, 5j8l, 5jaw, 5jbd, 5jcj, 5jcl, 5jdj, 5jhz, 5jia, 5jjs, 3toy, 3ttw, 3ttx, 3twa, 3twb, 3tyq, 3tyv, 3u49, 3u4o, 3u7i, 3u8a, 3ub1, 3udf, 3ues, 3ug3, 3umv, 3un2, 3un3, 3un5, 5tnm, 5tnr, 5tns, 5tpr, 5tqn, 5tqo, 5tqp, 5tro, 5tz3, 5tza, 5tzz, 5u1z, 5u21, 5u24, 5u2n, 5u7d, 5u8w, 5u9p, 5ucj, 4mo4, 4mo5, 4moq, 4mpb, 4mqj, 4mup, 4mz4, 4mz7, 4n0p, 4n0r, 4n0w, 4n2x, 4n4p, 4n6v, 4n8c, 4n9c, 4n9d, 4n9e, 4naa, 4nbm, 4nf2, 4ng3, 4nhd, 4ni8, 4nk4, 4nr0, 4ns1, 4nur, 4o13, 4o15, 4o19, 4o1b, 4o1d, 4o8m, 4oa4, 4ogd, 4oj0, 4oj5, 4oj6, 4ok4, 4omf, 4one, 4oqd, 4p12, 4p13, 4p5a, 4pbq, 4pc4, 4pcg, 4pch, 4pdd, 4pdx, 4pex, 4yjk, 4ytt, 4yx9, 4yzo, 4z0y, 4z0z, 4z13, 4z3d, 4z6l, 4z6o, 4z6p, 4z6r, 4z9f, 4z9n, 4z9y, 4zac, 4zbb, 4zbt, 4zd6, 5yab, 5yap, 5yfc, 5yfd, 5yj0, 5yj2, 5yj7, 5yja, 5yq0, 5yq2, 5yrt, 5yu3, 5yzo, 5z2l, 5zai, 5zbd, 5zcp, 5zcq, 5zfx, 5zg3, 6ldy, 6lgb, 6lgd, 6lgh, 6lgi, 6lr3, 6m35, 6m81, 6m9r, 6mbj, 6mfu, 6mgg, 6mgy, 6mk6, 6mls, 6mo3, 6mpd, 6mpr, 6mx6, 6aon, 6aqp, 6are, 6arf, 6arg, 6arh, 6aur, 6aus, 6auv, 6auw, 6awi, 6axe, 6azn, 6b4o, 6b6w, 6ba5, 6bbl, 6bkx, 6boj, 6bru, 6bu6, 6bun, 6bzb, 6bzn, 6c0y, 6c4a, 6c7d, 6c7g, 6c7i, 6c80, 6c9b, 6ca3, 6cac, 6cbk, 6cbl, 6cbo, 6cc6, 6cd0, 4jtj, 4jui, 4k1w, 4k2m, 4k2s, 4k5m, 4k9n, 4kc7, 4kc8, 4kea, 4kfd, 4kfn, 4kfo, 4kg6, 4kgi, 4khm, 4knu, 4ko2, 4ko3, 5vt6, 5vun, 5vuo, 5vv0, 5vwo, 5vwq, 5w15, 5w4z, 5w5t, 5w8h, 5w8k, 5wgm, 5wsk, 5wx6, 5wxu, 5x2y, 5x2z, 5x30, 5x49, 5x6p, 5llo, 5lnr, 5lnu, 5loq, 5ltp, 5lu0, 5luc, 5lug, 5lv3, 5m14, 5m2g, 5m4q, 5m8z, 5m91, 5m92, 5maa, 5mc2, 5mc4, 5med, 5jkl, 5jkm, 5jn5, 5jow, 5jox, 5jrh, 5jv4, 5jwz, 5jy1, 5jyd, 5k0a, 5k2z, 5k3o, 5k45, 5k4g, 5k4y, 5k6a, 5kf0, 5kf6, 5kit, 4rje, 4rk0, 4rlf, 4rlq, 4rm2, 4rm3, 4rmn, 4rnl, 4rpb, 4rsc, 4rsm, 4rvu, 4rz4, 4s3j, 4tjw, 4tk0, 4tla, 4tmb, 4tmc, 4nc4, 4pgn, 4q9n, 4rit, 4toq, 4uf0, 4wcd, 4xdc, 4yh2, 4ze8, 5a13, 5cjh, 5efo, 5fav, 5gmz, 5hw1, 5ioj, 5jkk, 5klo, 5lky, 5mh3, 5nx8, 5on1, 5syu, 5tnl, 5koi, 5kq0, 5kq6, 5kqk, 5kqn, 5ksf, 5ksg, 5ksk, 5ku6, 5kwe, 5kze, 5kzh, 5l05, 5l2f, 5l43, 5l44, 5l8d, 5lb3, 5le5, 5le6, 5syv, 5syx, 5t2k, 5t2p, 5t5v, 5t7o, 5t9y, 5tds, 5tdu, 5tey, 5tgd, 5tl4, 5tma, 5tne, 5tnf, 5tng, 5tni, 5tnj, 5tnk, 6n0b, 6n17, 6n2c, 6n92, 6n93, 6n94, 6n96, 6n97, 6n9i, 6n9r, 6na3, 6na4, 6na5, 6nes, 6nfp, 6ng4, 6ngi, 6ngj, 6ngm, 6ngx, 5min, 5mkb, 5mkv, 5mld, 5msd, 5mse, 5msq, 5mst, 5msu, 5mwu, 5mzi, 5mzy, 5n0l, 5n2i, 5n4d, 5n8d, 5nab, 5nae, 5nag, 5nah, 5nay, 5nd5, 5nf4, 5nh4, 5nh5, 5nh6, 5nha, 5nhd, 5nhm, 5nmx, 5nnb, 5no8, 5nqu, 5o48, 5o4h, 5o4i, 5o4j, 5o79, 5o9a, 5o9d, 5o9f, 5oco, 5ocr, 5ocs, 5ogz, 5ohf, 5ohy, 5oi0, 5oi1, 5oiv, 5oiw, 5oj4, 5xb8, 5xde, 5xdg, 5xdq, 5xfv, 5xfw, 5xm3, 5xwv, 5xwz, 5xxm, 5xy4, 5xzt, 5y02, 5y1h, 5y1r, 5y1s, 5y1v, 5y3i, 5y52, 6cfq, 6cic, 6cid, 6ciu, 6cja, 6cjb, 6cut, 6cyn, 6cyq, 6cz7, 6cz8, 6cz9, 6cza, 6czy, 6czz, 6d36, 6d3a, 6d6b, 6d6w, 6day, 6gan, 6gbx, 6gd0, 6gdo, 6gex, 6gmo, 6gom, 6gpl, 6gqt, 6grr, 6guo, 6gvb, 6gxs, 6h0b, 6h1o, 6h21, 6h2q, 6hbe, 6hc6, 6hcu, 6v6z, 6vh9, 6vjd, 6vlf, 6vmu, 6vpq, 6vtm, 6vxt, 6wgw, 6wk2, 6wt2, 6wy4, 6wyz, 6wz8, 6wzk, 6x2e, 6xdk, 6xec, 6xp1, 6xr5, 5uea, 5unc, 5unl, 5uof, 5upp, 5uqc, 5uqs, 5usw, 5utm, 5uyt, 5v0w, 5v38, 5v53, 5v7g, 5v7n, 5va8, 5vbi, 5vm5, 5vnx, 1egv, 1epx, 1f1x, 1ftr, 1fxo, 1g1l, 1g87, 1gco, 1gd1, 1gqy, 1gte, 1h0h, 1hdu, 1hee, 1hfw, 1hg1, 1hl2, 1hl5, 1hm5, 1hm9, 1hx6, 1j2t, 1j31, 1j3z, 1j9q, 1jnr, 1jpz, 1jq3, 1jsl, 1jsr, 1k54, 1k56, 1km0, 1kqf, 1l9x, 1lcp, 1lk5, 1m0w, 3isa, 4mko, 5ucw, 6dhu, 6die, 6dih, 6djd, 6dje, 6djf, 6dji, 6djj, 6dur, 6dvh, 6dwd, 6dyy, 6dzx, 6e0v, 6e43, 6e8o, 6e97, 6ea6, 6eaa, 6ebz, 6eow, 6erk, 6etz, 6eu4, 6ezz, 6f2m, 6f3m, 6f3o, 6f4x, 6f5v, 6f77, 6f91, 6fdf, 6ffz, 6fg0, 6fjl, 6foy, 6fpg, 6frn, 6fur, 6fvz, 6fw0, 6fw3, 6fwb, 6fwc, 6fwh, 6fxj, 6fxq, 6g1u, 6g4b, 6g50, 6g7m, 6g7x, 6g9c, 6nlj, 6nll, 6nln, 6nlx, 6nsd, 6nu5, 6nub, 6nw5, 6nxb, 6nxc, 6nxg, 6o4c, 6o4e, 6o4i, 6o63, 6o7n, 6o7p, 6o95, 6ofu, 6oia, 6ojm, 6ok1, 6ond, 6op1, 6op3, 6or9, 6org, 6os5, 6owe, 6p3h, 6p73, 6p7l, 6p83, 6p84, 6p86, 6p88, 6p8a, 6p8k, 6pab, 6pac, 6pae, 6pbn, 6pbp, 6pc0, 6pcb, 6pfn, 6pg8, 6pht, 6pi1, 6pi5, 6pi6, 6pia, 6pk0, 6pn3, 6pn5, 6png, 6po0, 5zga, 5zjg, 5zl5, 5zla, 5zn6, 5zou, 5zow, 5zox, 5zoy, 5zoz, 5zp0, 5zp1, 5zp2, 5zp3, 5zp4, 5zp5, 5zp6, 5zp7, 5zp8, 5zp9, 6ixt, 6iyw, 6izh, 6j0p, 6j0u, 6j0y, 6j27, 6j8q, 6jcl, 6jkr, 6jow, 6jta, 6juw, 6jv4, 6jvc, 6jz4, 6jz6, 6jzs, 6k8s, 6kbw, 6kf7, 6kfm, 6klk, 6knh, 6kri, 6ksa, 6ktk, 6ktl, 6kxh, 6kyi, 6kyj, 6l0a, 6l0p, 6l0r, 6l1b, 6l3m, 6l8t, 5gnx, 5gud, 5gue, 5gx8, 5h6s, 5h6t, 5h80, 5h89, 5hj5, 5hjx, 5hk9, 5hkb, 5hnn, 5hoq, 5hq8, 5hsi, 5hum, 5hvi, 5hvl, 5hw0, 5zpb, 5zpc, 5zpd, 5zpe, 5zpf, 5zpg, 5zph, 5zpi, 5zpj, 5zpk, 5zpl, 5zpm, 5zpn, 5zpo, 5zps, 5zqj, 6a50, 6ac1, 6ad4, 6ao1, 5vq3, 5x8f, 5y77, 5zg4, 5zpa, 6aoc, 6cek, 6dfv, 6edw, 6g9u, 6hfz, 6ixm, 6ldw, 6myz, 6nkh, 6pa3, 6pp1, 6r5i, 6s3x, 6tlk, 6v66, 6xub, 4wct, 4wdz, 4whq, 4wi1, 4wnn, 4wnq, 4wq6, 4wsj, 4wum, 4wvf, 4wz5, 4x0y, 4x16, 4x1c, 4x8b, 4x8e, 4xb6, 4xcw, 4xda, 2zwa, 2zwn, 2zya, 2zzd, 2zzs, 2zzx, 3a0k, 3a15, 3a2v, 3a77, 3a9r, 3a9s, 3afi, 3afn, 3ahn, 3ahy, 3ain, 3aio, 3an1, 3aov, 3ath, 3awd, 3axm, 3azy, 3b00, 3b1d, 3b1e, 3b1q, 3b37, 3b8l, 3bbc, 3bbf, 3ben, 3bf3, 3bg6, 3blz, 3bmo, 3bmq, 3dhf, 3dhi, 3dho, 3djd, 3dje, 3dnt, 3dsi, 3dsj, 3dx0, 3e3m, 3e49, 3e5w, 3e6q, 3ebh, 3ecd, 3ecj, 3eck, 3ed4, 3ede, 3eo8, 3etn, 3f0y, 3f4v, 3f98, 3ff1, 3fj1, 3fmc, 3fnm, 3fsu, 3fzn, 3g0t, 3g9k, 3ge4, 3giq, 3gke, 3gl0, 3gm6, 3gn2, 4zfj, 4zfl, 4zfz, 4zjz, 4zk1, 4zkw, 4zkx, 4zlu, 4zoc, 4zoe, 4zoz, 4zqr, 4zsw, 4zsy, 4zu4, 4zul, 4zw3, 4zw5, 5a04, 1m3u, 1m6s, 1m7s, 1m9x, 1mg0, 1mi3, 1mo9, 1mr7, 1ms1, 1ms3, 1mty, 1naa, 1nbu, 1ndb, 1npy, 1o58, 1o6i, 1o7t, 3ls3, 3lsa, 3lsc, 3lsm, 3lum, 3lv4, 3lvf, 3lvu, 3lxz, 3ly1, 3m0v, 3m0x, 3m2v, 3m5v, 3m8z, 3m9u, 3mbi, 3mcv, 3mfl, 3rrs, 3ru6, 3rwa, 3rwb, 3s0c, 3s1v, 3s1w, 3s1x, 3s2e, 3s5i, 3s7w, 3s81, 3s9y, 3sbq, 3scr, 3sfw, 3sgh, 3sjl, 3ska, 3slh, 3ssa, 3sx1, 3t3w, 3t5t, 3t67, 3t8i, 3t8v, 3td4, 3tfq, 3tgw, 3thc, 3thd, 3thu, 3tiv, 3uuw, 3v4n, 3v8h, 3vav, 3vc3, 3vgw, 3vkj, 3vpb, 3vpg, 3vqt, 3vs8, 3vwx, 3vyg, 3vz3, 3vzp, 3w5m, 3w5n, 3w6l, 3w8z, 6hg8, 6hkv, 6hlm, 6hm2, 6hnu, 6hqd, 6hqf, 6hsu, 6hti, 6htt, 6hu0, 6hu3, 6hzg, 6i0z, 6i4p, 6i4s, 6icn, 6ihi, 6ite, 6ixl, 5cpu, 5crf, 5cvj, 5cvu, 5d05, 5d08, 5d4i, 5d4v, 5d6o, 5dkv, 5dky, 5dlk, 5dna, 5do8, 5dtk, 5du7, 5dw3, 5dw4, 5dw5, 5dx6, 5dy9, 5e2h, 5ecn, 5ehr, 5ej4, 5ej9, 5eja, 5ejm, 5enq, 5eph, 5epm, 5evi, 5eyu, 5f0v, 5f2l, 5f3b, 5f4s, 5f4w, 5f52, 5f7j, 5f7x

**S2. Determination of the size of trimming box**

The dataset for training the constructed neural networks comprised the distribution patterns of the protein atoms around each hydration water molecule. To determine the appropriate size of the trimming box composed of 0.25×0.25×0.25 Å^3^ voxels for collecting the distribution patterns, we calculated the frequency distributions regarding the distances between the targeted hydration water molecules and surrounding protein atoms/hydration water molecules (Fig. S1). Because the tail part in the frequency distribution of carbon atoms reached 4.5 Å, the trimming box should have a minimum dimension of 9×9×9 Å^3^. In addition, to add 0.5 Å margins and set a water molecule at the center voxel of the box, we set the size of the scanning box to 10.25×10.25×10.25 Å^3^.

**
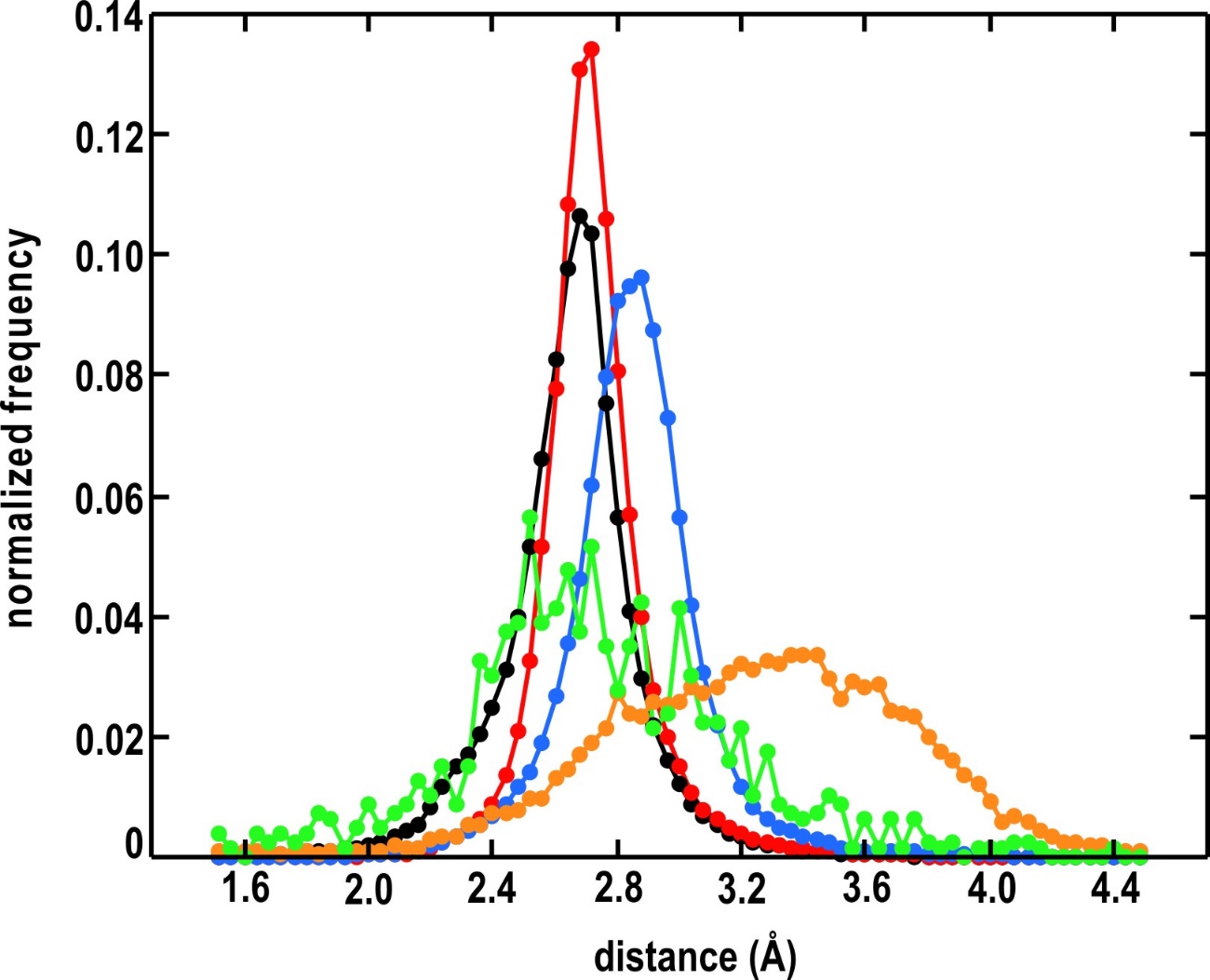
**

**Figure S1**

Normalized frequency distributions on 1,369,207 water-water (black line and symbols), 931,260 water-oxygen atom (red), 255,023 water-nitrogen atom (blue), 26,043 water-carbon atom (orange), and 799 water-sulfur atom (green) distances from 2145 crystal structures. Each frequency distribution counted at 0.04 Å step is normalized with respect to the total number of frequencies.

**S3. Optimization and selection of neural networks**

*Optimization of constructed six neural networks*

We constructed six neural networks (NNs) by varying the sizes of the convolution filters (CFs) (3×3×3 or 5×5×5), the number of convolution units (CUs) (2, 3, or 4), the number of channels in each block (4 or 32), and the number of layers (1, 3, or 4) in the fully connected block (FCB) (Table SI1). The number of nodes in each layer of the fully connected block was fixed at 32. The architectures of the six NNs are summarized in Table SI1.

As described in the main text, the NN model was first optimized using the coarse dataset to predict hydration probability distributions at a resolution of 0.50×0.50×0.50 Å^3^ voxels (designated coarse NN). In addition, the NN model was optimized independently using the fine dataset at a resolution of 0.25×0.25×0.25 Å^3^ voxels (fine NN). The feasibility of the six coarse NNs and the six fine NNs was evaluated for the accuracy and loss scores of the training and validation datasets, and the prediction of test crystal structures. As test structures, we used the crystal structures of glutamate dehydrogenase (GDH) and nitrile hydratase (NHase), which were excluded from the dataset (Table SI2).

*Selection of NN suitable for hydration prediction*

Among the six coarse NNs (Table SI1), the loss score of Model 1 was nearly 0.5 for the validation dataset, indicating failure of the optimization. In contrast, the other five coarse NNs (Models 2–6) were successfully optimized, as indicated by the accuracy scores in the range of 0.84–0.87, and the loss scores of 0.30–0.37 for the validation dataset. Among the five models, Model 6 displayed the best performance with respect to all the scores.

Regarding the six fine NNs, the optimization of Model No.1 failed again, but the other fine NNs were successfully optimized. In particular, Models 3, 5, and 6 gave nearly the same high accuracy scores (0.87) and low loss scores (0.29) for the validation dataset. However, it was unclear which of the three fine-NNs was the most feasible for predicting hydration probability distributions. We then compared the frequency distributions of the hydration probability at the hydration sites found in the crystal structures of GDH and NHase. It should be noted that the ideal hydration probability is 100% at the experimentally identified hydration sites. Figure SI2 compares the frequency distributions given by the fine-NNs of Models 2– 6. The frequency distributions for the first-layer hydration sites predicted by Models 2 and 3 had maxima at 90%–95% probability for both GDH and NHase. Model 5 had maxima at 98%, but the profile was similar to that of Model 3. Models 4 and 6 yielded monotonously increasing profiles. As a result, regarding hydration probability at hydration sites, Models 4 and 6 were suitable for predicting hydration structures. When inspecting the accuracy and the loss scores for the validation dataset and the GDH (Table SI1), Model 6 showed better scores than Model 4 for both coarse and fine prediction of hydration probability. Therefore, we selected Model 6 for hydration prediction in this study.

*Parameters affecting the prediction efficiency*

Here, we discuss the causes of the differences in the validation and loss scores of the six NNs. In principle, as each convolution layer (CL) reduces the amount of input data, the amount of output data from the convolution block (CB) depends on the number of CLs and pooling layers (PLs) included in the CB.

Regarding CLs, for instance, when the size of a CF is set to 5×5×5 in Models 1, 2, and 3, the first convolution operation reduces the size of the input data by 4×4×4. As one CU includes two CLs, in Model 1, the net size of the input data is reduced to 41×41×41 → 33×33×33 → 22×22×22 → 14×14×14 → 6×6×6 through the eight convolution layers included in the four CUs. In Model 2, the net size of the data was reduced to 41×41×41 → 33×33×33 → 22×22×22 → 14×14×14 through the six CLs included in the three CUs. In Model 3, the net size of the input data was reduced to 41×41×41 → 33×33×33 → 22×22×22 through the four CLs. When the size of CF is 3×3×3, as in Models.4,.5, and 6, which are composed of two CUs, the size of the data is reduced to 41×41×41 → 37×37×37 → 33×33×33.

Based on the examples described above, the failure in the optimization of Model No.1 indicates that 6×6×6 data yielded by the four CLs with 5×5×5 CF was insufficient to express the distribution of protein atoms to be hydrated. However, in Models No.2 and No.3, which had smaller numbers of CUs than Model No.1, the optimization of the NNs succeeded. Therefore, 14×14×14 output data from the CB are necessary.

In addition to CLs, as PL downsamples the input data, the number of CUs also influences the training. In the constructed NN, we used a pooling box of 2×2×2 voxels, which selected the maximum value of data in a 2×2×2 voxel and down-sampled to one voxel. Therefore, a larger number of PLs in the CB results in a smaller amount of output data.

Here, we show how the data are reduced by a set of PLs. For instance, by successively connecting four PLs and simulating the CB without CLs in Model 1, the amount of data is reduced in the order of 41×41×41→ 20×20×20 → 10×10×10 → 5×5×5 → 2×2×2. In the case of three successively connected PLs, simulating the CB without CLs in Model 2, the amount of data was reduced in the order of 41×41×41 → 20×20×20 → 10×10×10 → 5×5×5. In the case of two successively connected CUs simulating the CB without CLs in Model 3–6, the amount of data is reduced in the order of 41×41×41 → 20×20×20 → 10×10×10.

Based on the pooling size, the number of output data from the CB is 2×2×2 in Model 1 with four CUs, 5×5×5 in Model 2 with three CUs, and 10×10×10 in Model 3–6 with two CUs. The present results suggest that these two CUs are suitable for this prediction.

In addition to the size of CF and the number of PLs, the number of channels in the CL and the number of layers in the FCB influence the prediction efficiency to a lesser extent. When comparing the accuracy scores and the loss scores for the training dataset and the validation dataset between Models 4 and 5, which differed from each other only in the number of channels, the scores were better in Model 5. Therefore, 32 channels yielded better scores than four channels. The influence of the number of layers in the FCB was inspected by comparing the accuracy score and loss score of Models 5 and 6. When each layer had 32 nodes, the training results demonstrated that the loss score of Model 6 with one layer was lower than that of Model 5 with three layers.

**Table S1**. Comparison of the performance among the constructed NNs.

| Constructed NN Models | 1 | 2 | 3 | 4 | 5 | 6 |
| --- | --- | --- | --- | --- | --- | --- |
| Composition |  |  |  |  |  |  |
| Convolution block |  |  |  |  |  |  |
| Number of convolution units | 4 | 3 | 2 | 2 | 2 | 2 |
| Size of convolution filter | 5×5×5 | 5×5×5 | 5×5×5 | 3×3×3 | 3×3×3 | 3×3×3 |
| Number of channels | 4 | 4 | 4 | 4 | 32 | 32 |
| Fully connected block |  |  |  |  |  |  |
| Number of layers except the first input | 4 | 3 | 3 | 3 | 3 | 1 |
| Number of nodes | 32 | 32 | 32 | 32 | 32 | 32 |
| Number of parameters to be optimized | 20,322 | 30,234 | 138,226 | 131,954 | 1,112,738 | 1,110,626 |
| Performance of coarse NN |  |  |  |  |  |  |
| Training time (s) | 103 | 117 | 129 | 142 | 116 | 148 |
| Accuracy for training dataset | 0.5032 | 0.8439 | 0.8636 | 0.8653 | 0.8771 | 0.8693 |
| Loss for training dataset | 0.6932 | 0.3612 | 0.3302 | 0.3201 | 0.3024 | 0.3030 |
| Accuracy for validation dataset | 0.4910 | 0.8438 | 0.8481 | 0.8591 | 0.8677 | 0.8711 |
| Loss for validation dataset | 0.6934 | 0.3737 | 0.3430 | 0.3148 | 0.3037 | 0.3014 |
| Accuracy for test dataset (GDH) | --- | 0.8624 | 0.8680 | 0.8819 | 0.8808 | 0.8932 |
| Loss for test dataset (GDH) | --- | 0.3425 | 0.3054 | 0.2745 | 0.2718 | 0.2527 |
| Computational time for test data (GDH) (s) | 334 | 326 | 325 | 260 | 889 | 893 |
| Performance of fine NN |  |  |  |  |  |  |
| Training time (s) | 100 | 236 | 219 | 103 | 296 | 306 |
| Accuracy for training dataset | 0.5043 | 0.8525 | 0.8807 | 0.8737 | 0.8903 | 0.8763 |
| Loss for training dataset | 0.6931 | 0.3490 | 0.2917 | 0.2977 | 0.2690 | 0.2944 |
| Accuracy for validation dataset | 0.4799 | 0.8708 | 0.8796 | 0.8667 | 0.8733 | 0.8765 |
| Loss for validation dataset | 0.6939 | 0.3047 | 0.2856 | 0.3070 | 0.2890 | 0.2868 |
| Accuracy for test dataset (GDH) | --- | 0.8882 | 0.9014 | 0.8924 | 0.8992 | 0.8981 |
| Loss for test dataset (GDH) | --- | 0.2656 | 0.2333 | 0.2523 | 0.2468 | 0.2358 |
| Computational time for test data (GDH) (s)^a)^ | --- | 5415 | 5388 | 5504 | 9667 | 8822 |

^a)^ The calculation was performed for the candidates suggested in the prediction by the coarse NN.


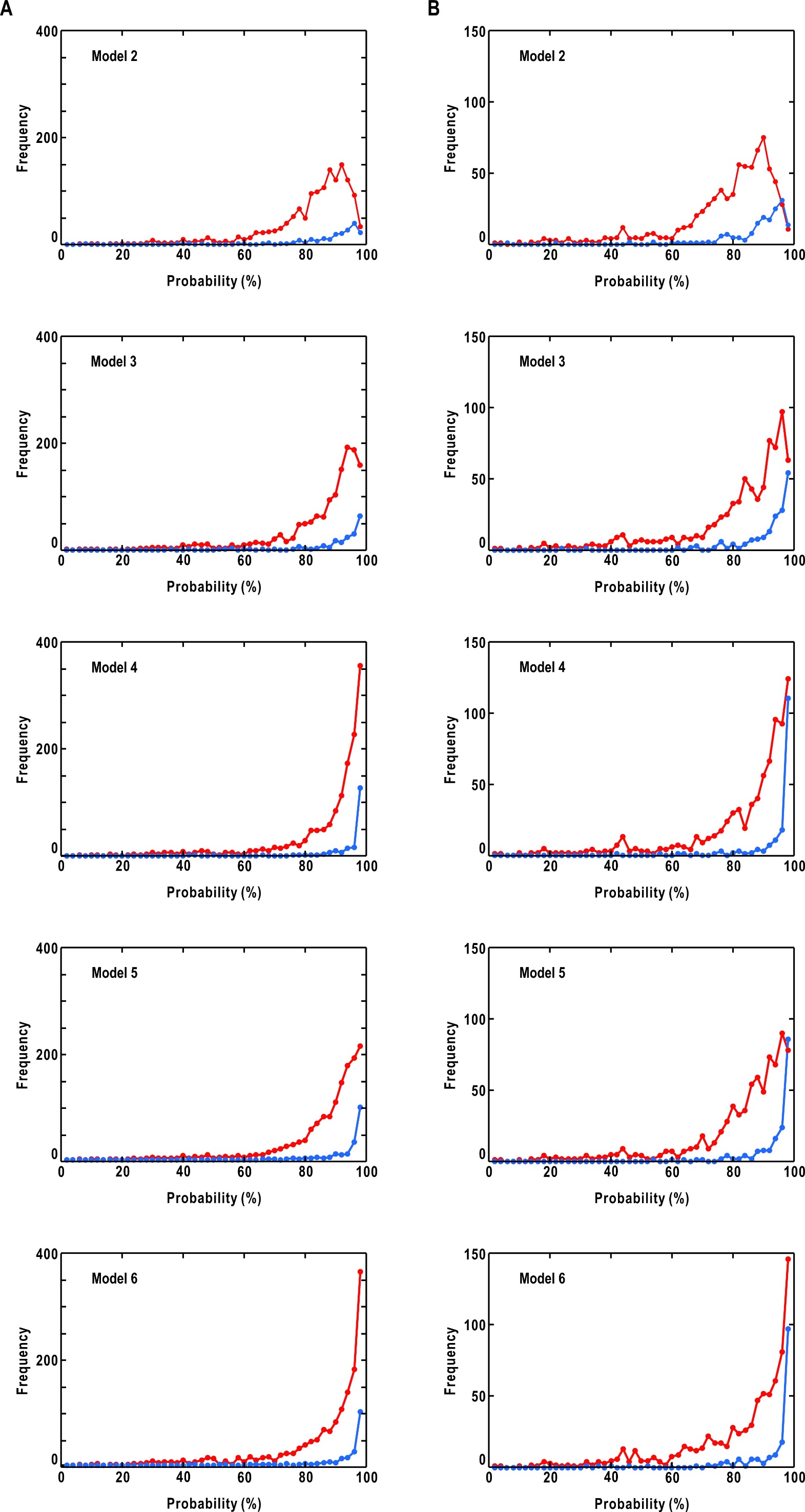


**Figure S2**

Frequency distributions of hydration probabilities predicted by fine-NN Models 2– 6 at experimental hydration sites. (**A**) The predicted hydration probability for hydration sites identified in the crystal structures of GDH. (**B**) The predicted hydration probability for hydration sites identified in the crystal structure of NHase. In each panel, the red and blue symbols/lines are the frequencies of the hydration probability for the crystal-water sites in the first-layer class and the inside class, respectively.

**S4. List of the Protein Data Bank accession codes of 300 crystal structures used for the extra validation of the selected neural network.**

1e5p, 1gve, 1gx5, 1jy1, 1kqp, 1nc7, 1nsc, 1nxu, 1rqp, 1tc1, 1usi, 1vpm, 1w5r, 1xgs, 1xrf, 1yx1, 2a2c, 2a50, 2axq, 2bkx, 2cd9, 2cxx, 2e5f, 2e9y, 2fm6, 2h6f, 2h8g, 2hhp, 2ixc, 2nxv, 2o3s, 2ob0, 2oem, 2p42, 2pbd, 2pbk, 2pwo, 2qb7, 2qzu, 2r6u, 2r7g, 2rdh, 2rmc, 2uvo, 2uzj, 2vbk, 2ve8, 2vnv, 2vve, 2w5v, 2w91, 2xxl, 2yh6, 2yp6, 2zyj, 3a2q, 3b5t, 3b8x, 3bje, 3bkw, 3bl9, 3cai, 3cb0, 3dss, 3dup, 3e9k, 3erp, 3euo, 3f8x, 3fpf, 3fwy, 3fxa, 3g7n, 3go9, 3gxg, 3h5k, 3hdo, 3hj4, 3hx8, 3imo, 3irs, 3l8h, 3m84, 3mil, 3n1f, 3nok, 3o14, 3oa3, 3oti, 3pu9, 3q34, 3qp8, 3rtl, 3t7h, 3u2u, 3w0k, 3w5s, 4a35, 4adn, 4ap9, 4au0, 4b1v, 4c12, 4c1l, 4c2v, 4cfq, 4chi, 4cnn, 4cog, 4dja, 4eiv, 4el4, 4es8, 4fai, 4fzl, 4ha4, 4ipu, 4iqy, 4itx, 4jdy, 4jzw, 4k7c, 4l6h, 4la9, 4lg1, 4lip, 4m1d, 4m1u, 4n01, 4n4j, 4nt1, 4ntk, 4nu0, 4ob0, 4ob1, 4odo, 4of4, 4oh7, 4olt, 4onw, 4opw, 4oze, 4pbc, 4pow, 4r19, 4rk6, 4rl3, 4u6d, 4uaf, 4usi, 4v0v, 4w64, 4wlh, 4wu0, 4ww7, 4wzf, 4xem, 4xmr, 4xtv, 4yag, 4yor, 4yzt, 4yzz, 4z1d, 4zwv, 4zyb, 5a6o, 5axa, 5b0r, 5b4b, 5b68, 5b6d, 5bk9, 5bmn, 5bmt, 5chs, 5ctm, 5dm0, 5doh, 5dow, 5dvw, 5ej3, 5fis, 5fly, 5gmc, 5gng, 5gwf, 5h2d, 5h94, 5i2h, 5irc, 5ixo, 5jr2, 5k4b, 5k91, 5kds, 5kkq, 5l77, 5lp0, 5lsl, 5m10, 5m7y, 5m89, 5mbq, 5n9t, 5nmv, 5nny, 5nps, 5o1v, 5obt, 5ofz, 5ow0, 5owo, 5sym, 5t07, 5tl5, 5u6a, 5ufd, 5uy1, 5v0z, 5w82, 5xvr, 5ylw, 5yqw, 5zee, 5zu5, 6ad3, 6ae8, 6aek, 6ao3, 6b1z, 6b5k, 6bq6, 6bw9, 6c29, 6cd7, 6d7a, 6e4d, 6e6u, 6fc1, 6g85, 6g86, 6gtz, 6hyf, 6il9, 6iod, 6ipb, 6jyz, 6kqs, 6n1m, 6nq4, 6nx0, 6ofk, 6om4, 6ozb, 6p8p, 6qj6, 6qla, 6qvs, 6ryd, 6sq2, 6stw, 6stz, 6su3, 6syi, 6t6l, 6t85, 6tr3, 6u1r, 6vd1, 6xc0, 6xx1, 6y0u, 6z7a, 6z9k, 6zb8, 6zeg, 6zn7, 7at0, 7b2i, 7by1, 7crg, 7d6r, 7dg2, 7dv7, 7kos, 7kpu, 7kqq, 7lz2, 7m7n, 7ncu, 7ogj, 7otf, 7r86, 7rlm, 7se8, 7sne, 7ta4, 7wnn, 7yxg

**Table S2**. Statistics of the diffraction data and structure refinement of proteins used to examine the feasibility of the constructed neural network.

| protein | Nitrile hydratase  (NHase) | Glutamate dehydrogenase  (GDH) | Scytalone dehydratase  (SDH) F162A mutant |
| --- | --- | --- | --- |
| **Diffraction data** |  |  |  |
| Temperature (K) | 100 | 100 | 37 ^1)^ |
| Space group | *P*2_1_2_1_2 | *P*2_1_ | *P*2_1_ |
| Asymmetric unit | (αβ)_2_ hetero-tetramer | hexamer | trimer |
| Lattice constants *a*, *b*, *c* (Å), β (°) | 116.61, 145.10, 51.27 | 112.99, 163.70, 133.07, 113.46 | 72.64, 61.30, 72.62, 120.02 |
| Resolution range (Å) Overall/highest shell | 100.00–1.70 / 1.72–1.70 | 113.00–1.80 / 1.82–1.80 | 72.64–1.45/1.47–1.45 |
| Total reflections | 487,668 | 1,181,214 | 365877 |
| Unique reflections | 93,113 | 413,444 | 98206 |
| Completeness (%) ^a)^ Overall/highest shell | 95.8/87.3 | 97.4/94.9 | 99.7/100.0 |
| *I* /σ Overall/highest shell | 23.96/3.13 | 10.9/1.0 | 23.8/4.2 |
| *R*_merge_^I^ Overall/highest shell ^b)^ | 0.052/0.368 | 0.068/0.560 | 0.043/0.297 |
| **Structure refinement** |  |  |  |
| PDB ID of starting model | 1EUZ ^2)^ | 2AHJ ^3)^ | 1IDP ^4)^ |
| Resolution range (Å) | 47.00–1.70 | 68.04ー1.80 | 36.346–1.445 |
| Unique reflections (completeness %) | 85,532 (92.5) | 353,960 (89.48) | 90784 (97.08) |
| *R*-factor c)/ *R*_free_-factor ^d)^ for 5% of reflections | 0.1497/0.1933 | 0.1719/0.1906 | 0.1647/0.1932 |
| **Structure model** |  |  |  |
| Amino acid residues in subunits / N- and C-terminal residue numbers of subunit | α1: 2-204, β1: 1-212,  α2: 1-205, β2: 1-212 | 1-419 for each subunit | A: 7-159, B: 6-157,  C: 7-158 |
| Metal atom, and ions | Fe:2, NO: 2, Zn:2, Ca: 2 |  |  |
| Small molecules and ions | Glycerol: 5,  Sulfate ion: 16 | Sulfate ion: 41,  acetate ion: 14 |  |
| Hydration water molecule | 1,239 | 1,766 | 573 |
| Deviation from ideal structure |  |  |  |
| R.m.s.d. of bond length (Å) | 0.0117 | 0.0118 | 0.0146 |
| R.m.s.d. of bond angle (°) | 1.670 | 1.670 | 1.959 |
| Ramachandran statistics (%) ^5)^ |  |  |  |
| Preferred/ allowed / outlier | 95.75 / 3.03 / 1.21 | 94.75 / 3.91 / 1.33 | 95.77 / 2.58 / 1.64 |

^a)^ Number of observations/unique reflections.

^b)^ $R_{merge}^{I}\text{ = }\sum_{\mathbf{h}} \sum_{i} \left|\text{ }I_{i}\left(\mathbf{h}\right)\text{- }\left\langle I\left(\mathbf{h}\right)\right\rangle\right|\text{ }/\text{ }\sum_{\mathbf{h}} \sum_{i} I_{i}\left(\mathbf{h}\right)$ , where $I_{i}\left(\mathbf{h}\right)$ is the intensity of the *i*-th observation of reflection $\mathbf{h}$.

^c)^ $R\text{ = }\sum_{\mathbf{h}} \left|F_{\text{obs}}\left(\mathbf{h}\right)-F_{\text{calc}}\left(\mathbf{h}\right)\right|/\sum_{\mathbf{h}} F_{\text{obs}}\left(\mathbf{h}\right)$, where $F_{\text{obs}}\left(\mathbf{h}\right)$ and $F_{\text{calc}}\left(\mathbf{h}\right)$ are the observed and calculated structure amplitudes of reflection $\mathbf{h}$, respectively.

^d)^ The *R*_free_ factor ^6)^ was calculated for the 5% of unique reflections, which were not used in the structure refinement throughout.

Diffraction data were collected at the BL41XU beamline of SPring-8. Indexing, integration, scaling, and post-refinement were performed using HKL2000 ^7)^. Structural refinement of each structural model was performed using Refmac5 ^8)^ in the CCP4 suite ^9)^.

**References**

1. Nakasako, M., Sawano, M. and Kawamoto, M. (2002) An open-flow helium cryostat for cryogenic X-ray diffraction experiments. *Rev. Sci. Instrum.* **73**, 1318-1320
2. Nakasako, M., Fujisawa, T. M. Nakasako, T. Fujisawa, S. Adachi, T. Kudo, and S. Higuchi (2001) Large-scale domain movements and hydration structure changes in the active-site cleft in unligated glutamate dehydrogenase from *Thermococcus profundus* studied by cryogenic X-ray crystal structure analysis and small-angle X-ray scattering. *Biochemistry* **40***,* 3069-3079.
3. Nagashima, S., Nakasako, M., Dohmae, N., Tsujimura, M., Takio, K., Odaka, M., Yohda, M., Kamiya, N. and Endo, I. (1998) Novel non-heme iron center of nitrile hydratase with a claw setting of oxygen atoms. *Nat*. *Struct*. *Biol*. **5**, 347-351.
4. Motoyama, T., Nakasako, M. and Yamaguchi, I. (2002) Crystallization of scytalone dehydratase F162A mutant in the unligated state and a preliminary X-ray diffraction study at 37 K. *Acta Crystallogr.* D **58**, 148–150.
5. PROCHECK Laskowski, R. A., MacArthur, M. W., Moss, D. S. and Thornton, J. M. (1993) PROCHECK: a program to check the stereochemical quality of protein structure. *J. Appl. Crystallogr*. **26**, 283-291.
6. Brünger, A.T. (1992) Free R value: a novel statistical quantity for assessing the accuracy of crystal structures. *Nature* **355**, 472-475.
7. Otwinowski, Z. and Minor, W. (1997) Processing of X-ray diffraction data collected in oscillation mode. *Methods Enzymol*. **276**, 307-326.
8. Murshudov G.N., Vagin A.A., and Dodson E.J (1997) Refinement of macromolecular structures by the maximum-likelihood method. *Acta Crystallogr*. D **53**, 240-255.
9. CCP4 (Coolaborative Computational Project, Number 4) (1994) The CCP4 suite: programs for protein crystallography. *Acta Crystallogr.* D **50**, 760-763.


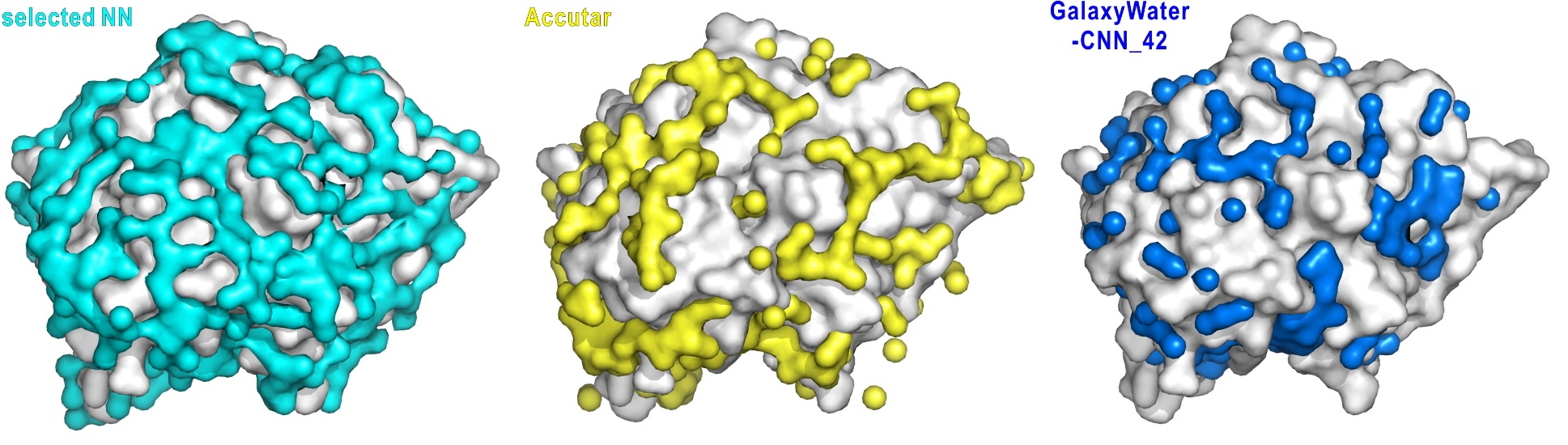


Figure S3.

Comparison of the predicted hydration sites among our NN and the two other NN-based methods. Distributions of the predicted hydration sites from our NN (cyan colored solid surface in the left panel), Accutar^1^ (yellow surface in the middle panel) and GalaxyWater-CNN_42^2^ (blue surface in the right panel) on the solvent accessible surface of apo-dethiobiotin synthase (the accession code of the Protein Data Bank: 1byi)^3^.

**References**

1. Huang, P., Xing, H., Zou, X., Han, Q., Liu, K., Sun, X., Wu, J. & Fan, J. Accurate prediction of hydration sites of proteins using energy model with atom embedding. *Front. Mol. Biosci*. **8**, 756075 (2021).
2. Park, S. & Seok, C. GalaxyWater-CNN: Prediction of water positions on the protein structure by a 3D-convolutional neural network. *J. Chem. Inf. Model.* **62**, 3157−3168 (2022).
3. Sandalova, T., Schneider, G., Kaeck, H. & Lindqvist, Y. Structure of dethiobiotin synthase at 0.97 A resolution. *Acta Crystallogr*. D **55**, 610-624 (1999).

**Table S3**. Validation scores of the three NN-based methods for protein structures used in the development of GalazyWater-CNN.

|  | Method | 1byi | 2a6z | 2fhz | 2fwh | 2ihj | 3vor | 6w63 | 6y84 | 6yb7 |
| --- | --- | --- | --- | --- | --- | --- | --- | --- | --- | --- |
| Coverage / % | Our method | 73.68 | 73.16 | 74.47 | 72.54 | 72.81 | 74.36 | 72.93 | 73.32 | 73.71 |
|  | Accutar^1^ | 44.53 | 49.41 | 43.08 | 39.50 | 47.67 | 43.68 | 43.90 | 44.46 | 42.34 |
|  | GalaxyWater-CNN_42^2^ | 46.46 | 46.49 | 51.73 | 46.06 | 48.00 | 50.04 | 48.74 | 49.22 | 50.12 |
| RMSD / Å | Our method | 1.22 | 1.09 | 0.97 | 1.09 | 0.95 | 1.07 | 0.98 | 1.06 | 1.07 |
|  | Accutar | 1.41 | 1.33 | 1.28 | 1.50 | 1.20 | 1.32 | 1.37 | 1.25 | 1.28 |
|  | GalaxyWater-CNN_42 | 1.49 | 1.32 | 1.06 | 1.40 | 1.17 | 1.26 | 1.11 | 1.22 | 1.20 |
| MAD / Å | Our method | 0.90 | 0.80 | 0.74 | 0.80 | 0.70 | 0.78 | 0.72 | 0.76 | 0.80 |
|  | Accutar | 1.02 | 0.91 | 0.87 | 1.06 | 0.81 | 0.91 | 0.95 | 0.85 | 0.86 |
|  | GalaxyWater-CNN_42 | 1.04 | 0.86 | 0.69 | 0.96 | 0.75 | 0.85 | 0.71 | 0.78 | 0.78 |

**References**

1. Huang, P., Xing, H., Zou, X., Han, Q., Liu, K., Sun, X., Wu, J. & Fan, J. Accurate prediction of hydration sites of proteins using energy model with atom embedding. *Front. Mol. Biosci*. **8**, 756075 (2021).
2. Park, S. & Seok, C. GalaxyWater-CNN: Prediction of water positions on the protein structure by a 3D-convolutional neural network. *J. Chem. Inf. Model.* **62**, 3157−3168 (2022).

**Table S4**. Computational times of the NN prediction of hydration probability distributions around proteins with different molecular weights.

| Protein | oligomeric state | number of residues | *M*_w_ | computation time (s) |
| --- | --- | --- | --- | --- |
| GDH | homo-hexamer | 2,514 | 282.18k | 9,706 |
| NHase | hetero-tetramer | 832 | 93.66k | 3,636 |
| F162A-mutated SDH | homo-trimer | 457 | 60.61k | 2,316 |
| AQP | subunit of tetramer | 251 | 30.04k | 1,251 |
| IL-1β | monomer | 153 | 17.40k | 382 |
